# Farm Fresh Foods for Healthy Kids (F3HK): An innovative community supported agriculture intervention to prevent childhood obesity in low-income families and strengthen local agricultural economies

**DOI:** 10.1186/s12889-017-4202-2

**Published:** 2017-04-08

**Authors:** Rebecca A. Seguin, Emily H. Morgan, Karla L. Hanson, Alice S. Ammerman, Stephanie B. Jilcott Pitts, Jane Kolodinsky, Marilyn Sitaker, Florence A. Becot, Leah M. Connor, Jennifer A. Garner, Jared T. McGuirt

**Affiliations:** 1 Division of Nutritional Sciences, Cornell University, Savage Hall, Ithaca, NY 14853 USA; 2 Department of Nutrition, Gillings School of Global Public Health & School of Medicine, University of North Carolina at Chapel Hill, Chapel Hill, NC 27599-8140 USA; 3 Center for Health Promotion and Disease Prevention, University of North Carolina at Chapel Hill, Chapel Hill, NC 27599-8140 USA; 4 Brody School of Medicine, East Carolina University, Lakeside Annex 8, Room 126, 600 Moye Boulevard, Greenville, NC 27834 USA; 5 Center for Rural Studies, University of Vermont, 206 Morrill Hall, Burlington, VT 05405 USA; 6 The Evergreen State College, 2700 Evergreen Pkwy NW, Olympia, WA 98505 USA; 7 Department of Community Development and Applied Economics, University of Vermont, Morrill Hall, Burlington, VT 05405 USA

**Keywords:** Childhood obesity, Fruit and vegetables, Nutrition, Diet, Community supported agriculture, Local food system, Local agriculture

## Abstract

**Background:**

Childhood obesity persists in the United States and is associated with serious health problems. Higher rates of obesity among children from disadvantaged households may be, in part, attributable to disparities in access to healthy foods such as fruits and vegetables. Community supported agriculture can improve access to and consumption of fresh produce, but the upfront payment structure, logistical barriers, and unfamiliarity with produce items may inhibit participation by low-income families. The aim of this project is to assess the impact of subsidized, or “cost-offset,” community supported agriculture participation coupled with tailored nutrition education for low-income families with children.

**Methods/design:**

The Farm Fresh Foods for Healthy Kids community-based, randomized intervention trial will build on formative and longitudinal research to examine the impact of cost-offset community supported agriculture on diet and other health behaviors as well as the economic impacts on local economies. The intervention will involve reduced-price community supported agriculture shares which can be paid for on a weekly basis, nine skill-based and seasonally-tailored healthy eating classes, and the provision of basic kitchen tools. Low income families with at least one child aged 2–12 years will be recruited to join existing community supported agriculture programs in New York, North Carolina, Vermont, and Washington. In each program, families will be randomized 1:1 to intervention or delayed intervention groups. Data will be collected at baseline, and in the fall and spring for 3 years. The primary outcomes are children’s intake of fruits and vegetables and foods high in sugar and/or (solid) fat, as well as diet quality; secondary outcomes include physical, behavioral, psychosocial, and environmental variables. Cost-effectiveness and economic impact at the farm and community levels also will be assessed.

**Discussion:**

This integrated project will provide important information and contribute to the evidence base regarding the use of local agricultural interventions to improve children’s dietary behaviors and weight maintenance. Findings also will inform the development of a toolkit for farmers and education modules related to local food system innovations for undergraduate and graduate students.

**Trial registration:**

ClinicalTrials.gov NCT02770196. Registered 5 April 2016.

**Electronic supplementary material:**

The online version of this article (doi:10.1186/s12889-017-4202-2) contains supplementary material, which is available to authorized users.

## Background

The prevalence of childhood obesity in the United States (US) remains high; approximately one third of children and adolescents are overweight or obese, placing a large health and economic burden on society [1–3]. Obesity early in life is associated with numerous serious health issues, including type 2 diabetes mellitus in youth and adulthood, obesity and associated chronic diseases in adulthood [4, 5], and premature death [5, 6]. Higher rates of obesity and poor health are evident in socially disadvantaged groups [7, 8] and disparities are growing among children and adolescents [9, 10].

A diet rich in fruits and vegetables provides important bioactive compounds and nutrients [11], is associated with decreased risk of cardiovascular disease, some cancers, and several other chronic diseases [12], and may help manage weight [13]. Despite the benefits of eating plentiful amounts of fruits and vegetables, most Americans, including children, do not meet dietary recommendations [14–16]. Substantial disparities in fruit and vegetable intake exist by income, region, and ethnicity [15], paralleling disparities in obesity and other nutrition-related diseases [7–10, 17–19]. Home availability is associated with children’s fruit and vegetable intake [20], and lower consumption by individuals in poor households [15, 21, 22] may be due, in part, to limited access to a variety of fresh, affordable foods [23]. Several US studies have found lower concentrations of supermarkets and shops selling fruits and vegetables in poor neighborhoods compared to wealthier neighborhoods [23]. Even when local food stores stock fresh produce, factors such as limited quantities, high prices, inferior quality, poor store cleanliness, and suboptimal item placement may be barriers to the purchase fruits and vegetables [24, 25].

Due to disparities in physical and financial access, there is growing interest in leveraging direct-to-consumer marketing of fresh produce through farmers’ markets and community-supported agriculture (CSA) to improve fruit and vegetable intake [26, 27]. CSA participants pay for a “share” of a farmer’s crop upfront, then benefit from consistent access to fresh produce throughout the growing season. The CSA shares can be significantly lower in cost than similar types and amounts of produce bought at the grocery store and CSA members often cite quality of produce among the top reasons for participation [28–32]. CSA members report that participation leads to an increase in the amount and variety of fruits and vegetables their households eat [29, 30, 32–35], an increase in meals consumed at home [33, 34], and a decrease in meals consumed away from home [29, 32, 34]. In a large cross-sectional study in Canada, buying from CSAs was associated with better diet quality and lower body mass index (BMI) and waist circumference [36]. However, few empirical studies of the impact of CSAs on dietary outcomes have used well-established measures such as 24-h dietary recalls and validated food frequency questionnaires [33, 35, 37].

From the farmers’ perspective, CSAs aim to provide a fair price, while covering the cost of production and labor [38, 39]. Potential economic benefits of the CSA model for farmers include improved financial security, decreased investments of time and money into marketing, and decreased production costs [40–43]. Yet the current evidence on economic outcomes is mixed. Some studies have found CSAs to be more profitable compared to wholesaling and farmers’ market distribution, although profitability depends on farm size and production costs [40, 44]. Other studies have found that many CSA farmers are dissatisfied with their economic returns and feel they do not receive adequate compensation for their work [38, 39, 45, 46]. These somewhat contradictory findings reflect the difficulty of gauging the profitability of the CSA model, because farmers may engage in several enterprises, and production and income fluctuate annually [39, 40, 44, 47]. Overall, research on CSA profitability is limited in scope and is based on small sample sizes. Beyond direct economic revenues for farmers, the CSA model and other direct marketing channels may have collateral economic and community benefits [48]. Policy makers and local food advocates have expressed strong interest in better understanding the wider economic impact of these local food initiatives [49], as evidenced by recent support from United States Department of Agriculture (USDA) through initiatives such as ‘Know your Farmer Know Your Food’.

While CSAs in the US have grown in number from an estimated 60 farms in 1990 [47] to closer to 4000 today [50], those who participate are more likely to have higher incomes [32, 51–53]. The standard requirement of a lump sum payment in advance of the growing season may inhibit participation by low-income families [54, 55]. While variations on the CSA model that offer cost-offset mechanisms to support low-income households are emerging, there is limited evaluation of these programs. We do know that low-income consumers who have participated in CSAs say that logistical barriers to share pick-up and unfamiliarity with CSA vegetables are barriers to sustained participation [55, 56].

Evidence suggests that supportive programming for buying and preparing local foods may help low-income families make use of produce marketed through direct-to-consumer channels. Hands-on activities like preparing and cooking food can provide vivid and motivating experiences that stimulate behavior change [57–61]. Further, interventions to improve cooking skills have demonstrated positive effects on dietary intake [61, 62]. While multiple reviews have found that providing produce discounts encourages individuals to consume more fruits and vegetables [56, 63–65], there is limited research on the effects of applying this model to CSAs, particularly when coupled with tailored education. One study found, compared to non-participants, an increase in the number of fruits and vegetables found in the homes of low-income families participating in a subsidized CSA program with nutrition education [56]. A similar study using farmers’ market coupons found that education had a positive impact on attitudes and beliefs about fruits and vegetables, while coupons had a positive effect on fruit and vegetable consumption [66].

There is a need for well-designed studies that evaluate the impact of agricultural programs, including CSAs, on diet and nutrition-related outcomes, especially for populations at elevated risk for malnutrition and nutrition-related diseases [37, 67, 68], and on farmers’ profitability. Thus, the aim of this project is to assess the impact of cost-offset community supported agriculture (CO-CSA) participation coupled with tailored nutrition education for low-income families with children in four geographically diverse states: New York (NY), North Carolina (NC), Vermont (VT), and Washington (WA). The overall goal is to examine CO-CSA participation as a strategy to improve dietary quality, help at-risk children achieve and maintain healthy body weight, and support vibrant local economies. The objectives are outlined in Table 1. We hypothesize that CO-CSA participation coupled with tailored nutrition education will increase access to healthier foods for low-income families, lead to behavioral changes that will prevent obesity among children and adolescents, and strengthen local agricultural economies.

**Table 1:** Project objectives

1. Examine whether CO-CSAs coupled with tailored nutrition education improve dietary intake and quality among children aged 2–12 in low-income households;
2. Examine the influence of CO-CSAs and tailored education on attitudes and behaviors related to nutrition, meal planning, and meal preparation;
3. Contrast CSA models to understand if and how variability in operational characteristics affect participation and intervention effectiveness in low-income families with children;
4. Estimate the economic impact of a CO-CSA program on the local economy;
5. Evaluate options for farmers to sustain the CO-CSA, and work with an advisory board, extension, and other stakeholders (e.g. CSA networks) to disseminate findings through development of a toolkit and related electronic resources to maximize impact; and
6. Develop and evaluate short-course modules and lectures for undergraduate and graduate students related to local food system innovations that are synergistic with the goal of obesity prevention and designed to enhance human capital relevant to U.S. agriculture.

## Methods

To achieve the objectives, this five-year project includes seven interconnected components: (1) formative research with stakeholders; (2) examination of dietary outcomes among current CO-CSA participants; (3) development of a skill-based, CSA-tailored, extension-delivered education curriculum; (4) randomized intervention trial of CO-CSA plus tailored education; (5) evaluation of economic impact for farmers and communities; (6) development of business plans for long-term sustainability of CO-CSAs; and (7) development of undergraduate and graduate-level education modules related to food systems and obesity prevention. This project is a transdisciplinary collaboration between researchers at Cornell University (coordinating center and NY performance center), University of North Carolina at Chapel Hill (NC performance center), University of Vermont (VT performance center), and The Evergreen State College (WA performance center). The research team possesses qualitative and quantitative expertise that spans nutrition, public health, rural development, and agricultural economics. A national advisory board will offer additional expertise related to sustainable agricultural business development and provide strategies to support broader generalizability.

All research activities involving human subjects have been reviewed and approved by the Institutional Review Boards (IRBs) at the University of Vermont and Cornell University (protocol ID #1501005266). The probability and magnitude of harm or discomfort anticipated in this research are not greater, in and of themselves, than those ordinarily encountered in daily life. Web conferencing will be used to train study personnel on the general research protocol and specific data collection techniques.

### Component 1: Formative research with stakeholders

To provide a broad perspective on the factors affecting the feasibility of CO-CSAs coupled with tailored nutrition education, formative evaluation involving key participant groups will be conducted in the first year of the study (Table 2). Methods used to collect data will include in-depth interviews, focus groups, quantitative assessment of consumers’ willingness to purchase CSA produce given certain pricing and accessibility scenarios, tabulation of the typical harvest schedule of CSA farmers, and surveys. The entire research team will provide input on the design of all data collection instruments. Data will be collected in all four states in Year 1. Individuals will have to speak English to participate. All participating adults (18+ years) will provide written or oral consent. For interviews with children, written consent from a legal guardian and child assent will be obtained.

**Table 2:** Participant groups and data collection methods to be used during formative research in New York, North Carolina, Vermont, and Washington

Study participants	Data collection method / tool to be administered
CSA farmers	
*CSA farmers with a CO-CSA*	• In-depth interviews covering farm and CSA operations, marketing strategies, customer profiles, most popular produce offerings, views on accepting SNAP/EBT, lessons learned, and experience using (or thoughts related to) the following options for subsidizing CSAs: work shares, sliding scales, donations, fundraising, grants, and revolving loans • Fruit and vegetable checklist regarding crops grown on their farm
*CSA farmers with no CO-CSA*	• In-depth interviews covering farm and CSA operations, marketing strategies, customer profiles, most popular produce, views on accepting SNAP/EBT, interest in implementing a CO-CSA, opinions on options for subsidizing CSAs, including work shares, sliding scales, donations, fundraising, grants, and revolving loans • Fruit and vegetable checklist regarding crops grown on their farm
Low-income households	
*Low-income adults*	• In-depth interview covering eating and cooking habits, perceptions of healthy foods, community supports for healthy eating, children’s preferences for types of fruits and vegetables, child snacking, tools needed to prepare produce, children’s involvement in food preparation, thoughts on local farms and farmers, knowledge of seasonal produce, CO-CSA program thoughts and preferences, and what would make it easier or more challenging to participate in a CO-CSA program • Choice experiment exercise testing willingness to purchase a CSA within hypothetical scenarios regarding variations in pricing, share frequency, and share variety [70] • Demographic survey, adapted from the BRFSS questionnaire [96] and the American Community Survey [95]
*Low-income children*	• Semi-structured interview covering fruit and vegetable familiarity and preferences, involvement in meal preparation, snacking habits, family and peer influences, nutrition knowledge and skills obtained through school or community, experiences with gardening and farms, and knowledge of seasonal produce
Full-paying CSA members	• In-depth interview covering views on features of their CSA, CSA preferences, factors that influence their participation, child involvement in CSAs, food shopping preferences, opinions on food assistance programming for limited income families, willingness to help offset the price of CSA shares, and thoughts on CSA cost-offset strategies including: SNAP use, workshares, donations, fundraising, and grants • Choice experiment exercise testing willingness to purchase a CSA within hypothetical scenarios regarding variations in pricing, share frequency, and share variety [70] • Demographic survey, adapted from the BRFSS questionnaire [96] and the American Community Survey [95]
Community health educators	• Online survey with questions about their professional experience and demographic characteristics, fruits and vegetables that are familiar and appealing to low-income families in their communities, their work related to local foods promotion, and nutrition education programs that their organizations offer to low-income families • In-depth interview covering types of nutrition education they provide, where they deliver the education, participants served, and to solicit suggestions for how CSA produce could be highlighted in the curriculum • Phone focus group covering how to best promote local foods, interest in teaching about local foods, thoughts on cost-offset CSA program, engaging low-income populations in nutrition education, identifying community organizations to partner with for this type of program, and recommendations and suggestions for sustainability

*Abbreviations*: *BRFSS* Behavioral Risk Factor Surveillance System; *CSA* Community Supported Agriculture; *CO*-*CSA* Cost-offset Community Supported Agriculture; *EBT* Electronic Benefits Transfer; *SNAP* Supplemental Nutrition Assistance Program

#### Data collection

##### Interviews with CSA farmers

In-depth interviews will be conducted across the four states with approximately 12 CSA farmers who operate a CO-CSA program and approximately 12 CSA farmers who are interested in CO-CSAs but who do not currently have a program in place. Farmers will be identified though internet searches and researchers’ networks and screened for eligibility over the phone. Topic guides will include questions about farm and CSA operations, marketing strategies, customer profiles, most popular produce offerings, views on accepting SNAP/EBT, and experience using or opinions on subsidizing CSA shares for lower income customers. Farmers who operate a CO-CSA program will also be asked about lessons learned. All participating farmers will be asked to complete a checklist of seasonal produce grown on their farms and indicate approximate time of harvest. Farmers will be compensated $50 for their time.

##### Interviews with adults and children in low-income households

In-person, in-depth interviews will be conducted with approximately 10 adults in low-income households in each state. Sampling will be focused on the communities in which the randomized trial (component 4) will be implemented, and will seek to include a balance of caregivers of children aged 2–7 years and those of children aged 8–12 years. Eligibility will include (1) being the primary caregiver of a child in the household aged 2–12 years; and (2) meeting an income threshold determined by being eligible for the Expanded Food and Nutrition Education Program (EFNEP) or being the primary caregiver of a child who receives free or reduced-price school lunch. Participants will be recruited using a variety of strategies, including flyers, emails, and in-person recruitment at community locations (e.g. social service departments). A screening form will be administered to interested adults. Those with children aged 8–12 years will be asked if they are willing to let their child participate in a simultaneous, but separate interview.

At the time of the interview, low-income adults will complete a brief demographic survey to document their age, sex, race/ethnicity, and the number of adults and children living in the household. The interview guide will cover eating and cooking habits, perceptions of healthy foods, community supports for healthy eating, children’s preferences for types of fruits and vegetables, child snacking, tools needed to prepare produce, children’s involvement in food preparation, thoughts on local farms and farmers, knowledge of seasonal produce, CO-CSA program thoughts and preferences, and what would make it easier or more challenging to participate in a CO-CSA program.

Immediately following the interview, an adaptation of a stated preference non-market evaluation econometric technique, commonly known as a “choice experiment” [69], will be utilized to determine individual preferences for CSA shares in hypothetical market situations. This adapted method previously has been used to examine willingness to shop at a farmers’ market [70]. The goal is to understand a participant’s stated preference for purchasing a CSA given variations in specific price, amount, variety, frequency, and distance values. Each factor will be examined individually and in combination, as food shopping conditions are often multi-factorial. Participants also will be asked about ideal values for each factor, as well as the particular produce items and amount of each of those items they would want in their CSA share. Lastly, preferences for purchasing a CSA versus shopping at a supermarket will be determined given hypothetical cost and travel time differences.

In each state, 5 children will take part in semi-structured interviews to explore fruit and vegetable familiarity and preferences, involvement in meal preparation, snacking habits, family and peer influences, nutrition knowledge and skills obtained through school or community, experiences with gardening and farms, and knowledge of seasonal produce. Child interviews will include photo elicitation [71] and a modified draw and write activity [72], where children will be asked to draw a meal and snack that they usually make for themselves and describe what they are drawing. Adults will be compensated $25 for their time and participating children will be compensated $5.

##### Interviews with full-paying CSA members

In-person, in-depth interviews will be conducted with 20 full-paying CSA members, who purchase a share from a farmer who is interviewed as part of the formative evaluation (component 1) or a farmer who will partner in the randomized trial (component 4). In each state, we will seek to include CSA members of at least two different farms in the sample, with a balance of individuals without children and those with children aged 2–12 years. The interview guide will cover views on CSA features, CSA preferences, factors that influence participation, child involvement in CSAs, food shopping preferences, opinions on food assistance programming for limited-income families, willingness to help offset the price of CSA shares, and thoughts on various CSA cost-offset strategies. Participating full-pay CSA members also will be asked to complete the same demographic survey and choice experiment outlined above. They will be compensated $20 for their time.

##### Interviews and focus groups with community health educators

In each state, 5 community health educators will be recruited via emails and direct contact to take part in the formative research. For each participant, there will be three sources of data: (1) an online questionnaire including multiple choice and open-ended questions; (2) an individual, in-depth phone interview; and (3) a phone-based focus group discussion. The online questionnaire will include the same demographic questions asked of participating low-income adults and full-paying CSA members, as well as questions about professional experience, work related to local foods promotion, fruits and vegetables that are familiar and appealing to low-income families in their communities, and nutrition education programs that their organizations offer to low-income families. The interview guide will include questions about the types of nutrition education they provide, where they deliver the education, participants served, and how they suggest CSA produce could be highlighted in the curriculum that will be developed as part of component 3 (curriculum development). Follow-up phone focus group discussions, each including educators from at least two states, will be held to extend findings from the questionnaire and interviews and gather educators’ perspectives on how to engage low-income populations in nutrition education, recommendations for identifying community organizations to partner with for this type of program, and suggestions for sustainability.

#### Management and analysis of formative data

##### Qualitative analysis

All interviews and focus groups will be conducted by trained study personnel, audio-recorded, transcribed verbatim, and imported into qualitative data management software for analysis. Initially, one researcher will read and code each transcript by question, and conduct rapid analysis to inform the intervention. Subsequently, two researchers will read each transcript, create and apply an initial codebook based on the questioning structure to a subset of the data. Following this, researchers will meet to discuss emerging themes, check inter-coder reliability, resolve discrepancies, and create a consensus codebook. Data then will be recoded using the revised codebook, and key themes will be reviewed and discussed.

##### Choice experiment analysis

In addition to the audio-recording, the choice experiment exercise completed by low-income adults and full-paying CSA members will be detailed with hand-written notes, which will be used to inform the development of the coding schema. Each participant will be assigned a “willingness to shop or buy” score based on number of times they said “yes” to each purchasing situation. Frequencies and percentages of participants willing to utilize CSAs for each price/accessibility situation will be generated. Nominal variables reflecting produce preferences, given frequency, size, and price, also will be tabulated. Results will be examined separately by race/ethnicity, geographic region, and household size. Researchers will meet to resolve discrepancies between coding and tabulation.

### Component 2: Examination of dietary outcomes among current CO-CSA participants

To inform the randomized trial (component 4) and explore longitudinal diet patterns among CO-CSA families with children, we are partnering with the Northeast Organic Farming Association of Vermont (NOFA-VT) to conduct a longitudinal quantitative examination of dietary behaviors among applicants to their statewide CO-CSA Farm Share program, which offers a subsidy of up to 50% of CSA share costs. Subsidies are provided on a first-come, first-served basis to households whose self-reported income is ≤185% of the federal poverty level (FPL). Lists of applicants to the summer and winter Farm Share programs will be provided by NOFA-VT, and households with at least one child aged 2–12 years and with an email address will be added to the research panel. Sample size calculations indicated that sixty households are needed to observe significance of a change in consumption of one-third of a serving of vegetables, with 95% confidence, and 80% power.

#### Data collection and outcomes

Using an online questionnaire, we will measure and track CSA participation; household food security; the household’s use of federal food assistance programs; fruit and vegetable availability in the home; and intake of beverages, snacks, and fruits and vegetables (in general and season-specific) by the adult caregiver and one child aged 2–12 years at first contact. Basic information on participant characteristics also will be collected. To inform the intervention, a subset of households will be asked additional questions about the cooking tools they own and the costs associated with participating in a CSA, and will be invited to complete three non-consecutive dietary recalls (2 week days and one weekend day) for the child using the Automated Self-Administered 24-h (ASA24) system [73]. Beginning in Year 1, data will be collected quarterly.

#### Analysis

Summary statistics will describe CO-CSA participant characteristics, food access and use of food assistance, as well as experience with CSAs. Mean total fruit and vegetable intake among CO-CSA participants will be compared to age-sex specific recommendations, and to national averages, using paired t-tests. Intake of fruits and vegetables, and of snacks and sugar-sweetened beverages, across two points in time will be compared using paired t-tests. In-season and out-of-season consumption of particular fruits and vegetables will be contrasted using nonparametric tests.

### Component 3: Develop skill-based, CSA-tailored education curriculum

The development of a CSA-tailored, skill-based nutrition education curriculum will be informed by components 1 (formative research) and 2 (longitudinal analysis). A curriculum advisory committee composed of extension education representatives from each of the four states will provide input and feedback throughout the curriculum development process. Information from existing EFNEP and Supplemental Nutrition Assistance Program Education (SNAP-Ed) curricula will be adapted and integrated, as appropriate.

Social Cognitive Theory [74], the Dietary Guidelines for Americans [75], and the Physical Activity Guidelines for Americans [76] will guide the design of learning objectives, education strategies and activities. The overarching objectives of the education will be to: (1) address attitudes and beliefs about the value of consuming fruits and vegetables (outcome expectation); (2) improve skills and self-efficacy with respect to storing, preparing, and consuming CSA produce (self-efficacy); (3) reduce potential barriers to acceptance of CSA produce and develop strategies to substitute fruits and vegetables for more energy-dense foods (barriers); (4) increase skills and self-efficacy related to preparation of CSA produce using minimal solid fat and sugar (behavioral capacity); (5) provide opportunities for participants to observe peers demonstrating newly acquired skills and share new experiences via group discussion (observational learning/modeling); and (6) provide information and strategies to help families be more active in daily life and reduce sedentary time, particularly screen time.

The curriculum will be composed of nine lessons, with three relevant to the early CSA season, three relevant to the middle of the CSA season, and three relevant to the late CSA season. Lessons will be modeled using the 4A Approach [77]: a beginning “anchor” activity will ground participants’ learning in their current knowledge and behaviors related to the lesson’s topic; the educator will “add” to current knowledge by providing participants’ with information on and demonstration of new knowledge and skills; participants “apply” what they learn through hands-on activities; and a closing “away” activity will help participants plan how they will use what they have learned in their every-day lives. Most classes will include at least one “apply” activity involving food preparation given the evidence that such experiences may stimulate behavior change, particularly an increase in fruit and vegetable intake [61, 62]. A subset of classes will involve field-based learning (e.g. grocery store and farm tours). Although the education will focus on adults, each lesson will include child-friendly materials and activities. Supportive out-of-class materials also will be developed.

### Component 4: Randomized intervention trial of CO-CSA plus tailored education

Incorporating the findings and products generated in components 1–3 (formative evaluation, longitudinal analysis, and curriculum development), we will implement an exploratory field experiment – the Farm Fresh Foods for Healthy Kids (F3HK) intervention – beginning in Year 2. This multistate randomized intervention trial will target obesity prevention in low-income families through a transdisciplinary approach that improves access to affordable, local, seasonal produce through the provision of a CO-CSA share; supports dietary and other obesity-related behavior changes (e.g. reducing sedentary time) through tailored education to increase knowledge, skills, and self-efficacy; and provides increased revenue and business supports to CSA farmers, which will help support local agricultural economies.

The intervention will be implemented across communities in New York, North Carolina, Vermont, and Washington. A list of specific communities is available at ClinicalTrials.gov. Participants will receive a weekly CO-CSA share at 50% discount and the education curriculum developed in component 3 (curriculum development). The remaining 50% cost will be paid by the study to the farmers upfront. The CO-CSA will fit into existing CSA programs.

Each state will recruit 60 participants within at least two CSA programs. Participants will enroll in the CO-CSA for two seasons and participate in the CSA-tailored education during the first season. We will randomize families into either the intervention or control (delayed intervention) group. Group 1 (intervention) families will participate in the CO-CSA plus tailored education in Year 2 and Year 3; Group 2 (control) families will receive the same CO-CSA plus tailored education in Year 3 and Year 4 (Table 3). A delayed parallel design was selected in order to keep the benefits of participation, and therefore the motivation to keep participating, equivalent for parallel groups. We will work with participating farmers to support the transition to alternative funding for the cost-offset following completion of the intervention. Participants will be asked not to enroll in a CSA outside of the study. Participants may enroll in other nutrition education at any time during the trial. Data will be collected at identical time points for all participants. Bi-annual engagement postcards or letters will be mailed to all participants with the aim of increasing retention.

**Table 3:** Delayed intervention design for component 4 (randomized trial)

	Year 2	Year 3	Year 4
Group 1 – intervention	CO-CSA + Education	CO-CSA	Sustainability
Group 2 – control	Data collection only	CO-CSA + Education	CO-CSA
Group 3 – intervention		CO-CSA + Education	Sustainability
Group 4 – control		Data collection only	CO-CSA + Education

**Fig. 1:**
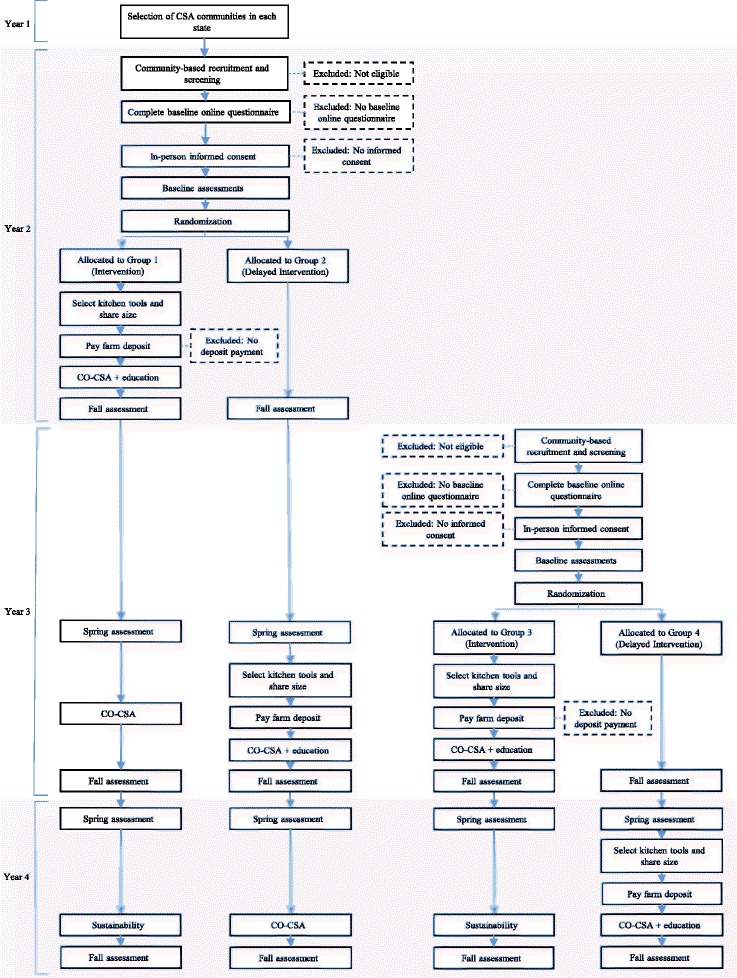
Flow of participants in component 4 (randomized trial) from recruitment through the end of the study

In Year 3, to compensate for study attrition, we will recruit additional participants and randomly assign into an identical intervention of shorter duration or control (delayed intervention). Group 3 (intervention) families will participate in the CO-CSA plus tailored education in Year 3 only; while Group 4 families will receive the same CO-CSA plus tailored education in Year 4 only. The study timeline is presented in Fig. 1.

#### Farms and educators

We will seek farms with an established CSA program who accept, or are willing to accept, Supplemental Nutrition Assistance Program (SNAP) benefits; are willing to offer weekly payment plans for study participants; and who agree to participate in the creation and implementation of a sustainability plan to keep the CO-CSA program in place at the conclusion of the intervention period. Additionally, there must be a professional community health educator (herein referred to as an educator) in the community who is able and willing to deliver the CSA-tailored curriculum. The educator will also assist the study team in recruiting and screening participants. Educators will have extensive experience working with low-income families and delivering nutrition programming and many will have an affiliation with cooperative extension (https://nifa.usda.gov/extension). Educators will complete an interactive, two-hour, web-based training on the CSA-tailored curriculum, covering the organization and content of the curriculum and a review of best practices for educating adult learners, including tenants of Dialogue-Based Adult Learning and Adult Learning Theory [78–80].

#### Participants

##### Recruitment

Study personnel and educators will recruit participants via flyers, newspapers, websites, and social media, as well as direct recruitment at schools, churches, libraries, community service organizations, local events, and “word of mouth.”

##### Screening and eligibility

Potential participants will be screened for eligibility using an electronic tablet-based screening tool. The eligibility criteria for participation is: (1) age 18 years or older; (2) English-speaking; (3) parent or legal guardian of at least one child between 2 and 12 years of age in the household (herein referred to as a caregiver); (4) meet income requirements of ≤185% of FPL (self-reported); (5) have not participated in a CSA in the past 3 years; (6) be willing to use their SNAP benefits or their own money to purchase the 50% CO-CSA share; (7) have an active email address and access to a computer or tablet for online data collection; and (8) be willing to attend CSA-tailored education classes as part of the intervention.

To enroll into the study, participants must: (1) attend a baseline assessment visit and provide written consent; (2) complete the first online questionnaire; and (3) if assigned to intervention, pay the farm a deposit equal to two weekly CO-CSA payments (refundable at the end of the season if all payments are made). The decision to require a deposit draws on the experiences of a program that provided no-cost CSA shares to low-income consumers in North Carolina, which indicate participants may be more engaged with a CSA arrangement if they make a financial commitment [55].

In each participating household, one caregiver and one child aged 2–12 years (herein referred to as the focal child) will enroll in the study. Caregivers will provide written informed consent to take part and written permission for the focal child to participate. Children 7–12 years will provide oral assent. Consent and assent will be obtained by trained study staff. Any ancillary studies that develop out of this project will obtain participant consent separately. Informed consent materials are available as Additional file 1.

#### Sample size calculations

We plan to test the hypothesis that there are equivalent mean changes in outcome variables in intervention and control groups. We estimated that a sample size of 240 households (120 intervention and 120 control) was needed to observe significance of a medium effect size (0.5 standard deviation), in clusters of 20 households within 12 CSA program communities (intra-class correlation of 0.05; 25% attrition from pre- to post-season, 95% confidence level, and power of 0.8). This strategy should allow us to detect as significant a difference between changes in fruit and vegetable intake of approximately one-third of a serving per day.

#### Randomization

Following enrollment and baseline assessment, participants will be 1:1 randomly assigned to intervention and control groups within CSA program communities by trained research staff using a computer-generated randomizer to assure equivalent group sizes at each farm location. Randomization will take place after informed consent is obtained at study enrollment appointments. Given the nature of the intervention, neither the participants nor the study staff will be blinded to group assignment.

#### Intervention components

##### CO-CSA

The shares offered through the CO-CSA program will be identical to standard summer CSA shares offered to full-paying CSA members. If the farm offers more than one size share (e.g. small share, standard share, large share), study participants will select their preferred option. Shares can be paid for at the start of the season or on a weekly basis at pick-up using cash, EBT, and another form of payment accepted by the farm. Participants will be sent reminder messages for the first 4 weeks of the CSA season.

##### Curriculum

Educators will deliver the curriculum developed in component 3 on consecutive weeks in three-lesson clusters during early, mid-, and late CSA season, with timing determined by performance centers based on the local harvest calendar. The classes will be scheduled for days, times, and locations that are convenient for most participants. Participants will be encouraged to attend all classes and will be reminded of the date and time of each session by the local educator. Handouts will be provided so that participants can share what they learn with non-attending family members and a password-protected project website will be developed where participants who are unable to attend classes will be able to access materials that align with lesson content, including lesson handouts and recipes that feature common CSA vegetables.

##### Kitchen tools

To help participating families prepare their CSA produce, free kitchen tools will be provided. Participants will be able to select two to four of the following items: food processor, crockpot, stockpot, large cutting board, chef’s knife, salad spinner, reusable grocery bag. Participants will choose kitchen tools just prior to their first year of CO-CSA participation. These kitchen tools will be mailed directly to participants’ homes. Additionally, a vegetable peeler, vegetable scrub brush, and paring knife will be distributed at the healthy eating classes for participants to keep.

##### Data collection and outcomes

We will assess changes from baseline in the quantity and variety of fruits and vegetables consumed, intake of energy-dense and processed foods, and dermal carotenoid levels for both children and adults. We also will measure estimated energy intake and dietary quality for children, food-related changes in the household environment (including household food security), caregivers’ knowledge and skills related to healthy eating, and children’s BMI percentile, physical activity, and sedentary behavior (Table 4).

**Table 4:** Primary and secondary outcome measures for component 4 (randomized trial)

Outcome	Measure	Source/Instrument
Primary outcomes		
Child’s fruit and vegetable intake	• Fruit and vegetable screener	• National Cancer Institute’s All-Day Fruit and Vegetable Screener [82]^a^
	• Three dietary recalls	• Automated Self-Administered 24-h dietary recall (ASA24–2016™) [73]
Child’s intake of sugar-sweetened beverages and processed snacks	• Beverage and snacks questionnaire	• Beverage and snack questionnaire 2 (BSQ2) [84, 85]^a^
	• Three dietary recalls	• Automated Self-Administered 24-h dietary recall (ASA24–2016™) [73]
Child’s caloric intake as a percent of estimated energy requirements	• Three dietary recalls	• Automated Self-Administered 24-h dietary recall (ASA24–2016™) [73]
Child’s dermal carotenoid levels	• Resonance Raman spectroscopy (RRS) of the palm	• Pharmanex Biophotonic Scanner S3 (NuSkin, Provo, UT)
Child’s diet quality	• Three dietary recalls	• Automated Self-Administered 24-h dietary recall (ASA24–2016™) [73]
	• Fruit and vegetable screener	• National Cancer Institute’s All-Day Fruit and Vegetable Screener [82]^a^
	• Beverage and snacks questionnaire	• Beverage and snack questionnaire 2 (BSQ2) [84, 85]^a^
Secondary outcomes		
Child’s BMI percentile	• Height and weight measured by trained research staff	• Lohman [81]
Child’s physical activity	• Questionnaire questions	• Burdette et al. [93] and Youth Risk Behavior Survey [92]^a^
Child’s sedentary behavior	• Questionnaire questions	• Youth Risk Behavior Surveillance System (YRBSS) questionnaire [92]^a^
Caregiver’s knowledge, attitudes, and beliefs about fruits and vegetables	• Questionnaire module	• Negative Cooking Attitude scale [86]^a^, Self-Efficacy for Eating/Cooking Fruits and Vegetables scale [86]^a^, General Nutrition Knowledge Belief score [87]^a^, and original
Caregiver’s ability to select, store, and prepare CSA produce	• Questionnaire module	• Cooking Techniques and Meal Preparation Self-Efficacy Scale [86]^a^
Caregiver’s ability to substitute fruit and vegetables for energy-dense foods	• Questionnaire module	• Original
Caregiver’s ability to prepare foods to minimize added (solid) fat and sugar	• Questionnaire module	• Original
Availability and accessibility of fruits and vegetables in the home	• Questionnaire module	• Scales for fruit and vegetable availability and accessibility within the home [88]
Caregiver’s fruit and vegetable intake	• Fruit and vegetable screener	• National Cancer Institute’s All-Day Fruit and Vegetable Screener [82]^a^
Caregiver’s intake of sugar-sweetened beverages and processed snacks	• Beverage and snacks questionnaire	• Beverage and snack questionnaire 2 (BSQ2) [84, 85]^a^
Caregiver’s dermal carotenoid levels	• Resonance Raman Spectroscopy (RRS) of the palm	• Pharmanex Biophotonic Scanner S3 (NuSkin, Provo, UT)
Household food security	• Questionnaire module	• US Department of Agriculture 6-item Food Security Module with 30-day reference period [89]^a^

^a^Indicates the tool was modified or adapted

The study team at Cornell will oversee all online questionnaire-based data collection, including dietary recalls. Study staff at each performance center will travel to the community sites to collect anthropometric and dermal measures. The schedule for data collection is shown in Table 5. Data collection instruments can be requested from the Principal Investigator (RAS).

**Table 5:** Data collection schedule: component 4 (randomized trial)

Assessments	Baseline	Fall	Spring
Informed consent form	**X**		
Physical measurements			
Anthropometric measures (child)	**X**	**X**	
Dermal carotenoid measures (caregiver and child)	**X**	**X**	
Dietary recalls (child)	**X**	**X**	
Online questionnaire modules			
Brief dietary measures (caregiver and child)	**X**	**X**	**X**
Fruit and vegetable knowledge attitudes, and beliefs (caregiver)	**X**	**X**	
Ability to select, store, and prepare CSA produce (caregiver)	**X**	**X**	
Ability to substitute fruit and vegetables for energy-dense foods (caregiver)	**X**	**X**	
Ability to prepare foods to minimize added (solid) fat and sugar (caregiver)	**X**	**X**	
Fruit and vegetable availability and accessibility within the home (household)	**X**	**X**	
Food security (household)	**X**	**X**	**X**
Nutrition assistance and education participation (household)	**X**	**X**	**X**
Physical activity and sedentary behavior measures (child)	**X**	**X**	**X**
General health status (caregiver and child)	**X**	**X**	**X**
Costs associated with food purchasing and preparation (household)	**X**	**X**	**X**
Costs associated with CO-CSA participation (household)		**X** ^**a**^	
Kitchen inventory (household)	**X**		
Household composition and demographics	**X**	**X**	**X**
CSA participation (outside of/in addition to the research study; household)	**X**	**X**	**X**
	Year 2	Year 3	Year 4
Process evaluation			
Program evaluation			
Dose delivered	**X**	**X**	**X**
Dose received	**X**	**X**	
Fidelity	**X**	**X**	
Experiences	**X**	**X**	
Program cost	**X**		
Non-participation	**X**		

^a^Subset of participants

##### Physical measures

Anthropometric and dermal measures will be collected at baseline and in the fall of Years 2–4.

**Anthropometric measures.** Anthropometric measures will be taken without shoes and in light clothing according to standard procedures [81]. Weight will be measured to the nearest 0.2 lbs. using the Seca Clara 803 scale. Height will be measured to the nearest 0.125 in. with the Seca 213 portable stadiometer. Weight and height measurements will be collected in duplicate unless the two measurements are more than 0.4 lbs. or 0.125 in. apart. In that case, a third measurement will be taken.

**Dermal carotenoid measures.** Dermal carotenoid levels will be assessed as an objective measure of fruit and vegetable intake. Study staff will use the Pharmanex Biophotonic Scanner S3 (NuSkin, Provo, UT), which non-invasively measures the concentration of carotenoids in skin tissues using resonance Raman spectroscopy, to scan a specific point of the palm of one hand. Dermal carotenoids will be measured in duplicate, unless the two measurements are greater than 2000 units apart. In that case, a third measurement will be taken. The same hand will be used at each assessment.

##### Dietary recalls

The National Cancer Institute’s Automated Self-Administered 24-h Recall system (ASA24–2016™) will be used to collect sets of three dietary recalls for the focal child [73]. This tool is based on the US Department of Agriculture’s interviewer-administered Automated Multiple-Pass Method (AMPM) and collects data on foods, drinks, and dietary supplements consumed the previous day. For children aged 2–5 years, the adult caregiver will complete the recall for the child. For children aged 6 years and older, the child will be asked to help the caregiver complete the recall.

##### Online questionnaires

Study participants will complete online questionnaires at baseline, in fall of Years 2–4, and spring of Years 3–4.

**Brief dietary measures.** Dietary intake of fruits and vegetables will be measured using the National Cancer Institute’s All-Day Fruit and Vegetable Screener (FVS) [82]. The FVS is a validated, 19-item instrument, which asks about the frequency and quantity of consumption of fruit and vegetables during the past month. We will adapt the FVS to an online format and add visual aids to improve portion size estimation [83]. Intake of sugar sweetened beverages and processed foods will be collected using the second version of the beverage and snack questionnaire (BSQ2) [84, 85]. The BSQ2 asks about frequency of consumption of beverages, savory snacks, and sweets in the last 7 days. To simplify the tool and reduce duplication with the FVS, we will remove questions about consumption of vegetables, fruits, or 100% fruit juice and will not ask participants to specify if foods were consumed in school or out of school. We also will ask whether eating habits in the past month were typical, the proportion of meals prepared and/or eaten together, and who completed the FVS and BSQ2 for the child.

**Fruit and vegetable knowledge, attitudes, and beliefs.** Caregivers’ *knowledge* of recommended fruit and vegetable consumption will be assessed by two ordinal variables, one with response choices in cups and the other relative to plate images. Caregivers’ *attitudes* toward food preparation will be captured by the Negative Cooking Attitude scale which sums ordinal responses to four negative statements about cooking [86]. The Self-Efficacy for Eating/Cooking Fruits and Vegetables scale [86] will be streamlined to focus only on items relating to fruits and vegetables, and record ordinal responses to four items. General nutrition *beliefs* will be assessed using the General Nutrition Knowledge Belief Score which sums ordinal responses to 11 healthy eating statements [87]. These beliefs include items directly relevant to F3HK, including the importance to participants of eating diets that include plenty of fruits and vegetables, use salt or sodium only in moderation, are low in saturated fat, and use sugars only in moderation.

**Caregiver’s ability to select, store, and prepare CSA produce.** The Cooking Techniques and Meal Preparation Self-Efficacy scale [86] assesses participant confidence in knife skills, preparation techniques, and basic cooking skills. We will expand the scale to include additional items that align with the aims and activities of the CSA-tailored education. Items on washing, storing, freezing, and drying produce will be added, as well as the preparation of leafy greens and cooking with herbs, onions, and garlic, which will be emphasized in the curriculum.

**Caregiver’s ability to substitute fruit and vegetables for energy-dense foods.** Caregivers will be asked about the monthly frequency of preparing nine specific fruit or vegetable-based snacks for their children (apple wedges, melon slices, plain berries, other fruits, carrot sticks, celery sticks, cucumber sticks, pepper slices, or other vegetables). The curriculum will emphasize substituting these particular foods for more energy-dense snacks.

**Caregiver’s ability to prepare foods to minimize added (solid) fat and sugar.** Caregivers will be asked about the monthly frequency of preparing for dinner those vegetables which are typically abundant in CSA shares (lettuce or other salad greens, green or red cabbage, other greens, potatoes, other root vegetables, and winter squash), and their most frequent preparation method for each (raw; steamed, boiled or baked; deep fat fried; roasted or sautéed in oil; cooked with meat, butter or cheese; or cooked another way).

**Fruit and vegetable availability and accessibility within the home.** Home fruit and vegetable availability will be assessed using questions from a 3-item scale, which asks caregivers “How often are the following true? 1) We have fruits and vegetables in my home, 2) In my home, vegetables are served at meals, 3) In my home, fruit is served for dessert” [88]. Home fruit and vegetable accessibility will be assessed using questions from a 4-item scale, which asks caregivers, “How often are the following true? 1) In my home, there is fruit available to have as a snack, 2) In my home, there is vegetable available to have as a snack, 3) In my home, there are cut-up vegetables in the fridge for my child to eat, and 4) In my home, there is fresh fruit on the counter, table, or somewhere else where my child can easily get to it” [88]. For both scales, four ordinal response options will be offered (hardly ever, rarely, sometimes, often). Caregivers also will be asked to report how easy or difficult it is to financially afford and physically access acceptable quality fruits and vegetables from five ordinal response options.

**Household food security.** Household food security will be classified by the 6-item short form of the USDA Food Security Survey Module (FSSM), with a 30-day reference period [89]. Items ask respondents about the conditions and experiences of food insufficiency due to resource limitations, and the behavioral responses of household members to these conditions. The form will be converted to an online format, but questions and response options will not be modified. Participants that affirm two or more items are considered to live in a food insecure household.

**Nutrition assistance and education participation.** Participation in school meal programs will be measured using seven questions from the National Health and Nutrition Examination Survey (NHANES) Diet Behavior and Nutrition Questionnaire [90]. Minor modifications will be made to simplify some questions and response options, and improve their suitability to an online format. We also will ask a follow-up question to determine whether the focal child’s school offers universal free breakfast. Three questions from the NHANES Food Security Module [91] will be used to assess participation in SNAP and the Special Supplemental Food Program for Women, Infants, and Children (WIC). These questions will be modified to specify a one-month reference period, rather than a one-year reference period. Study participants also will be asked if they participated in nutrition education classes other than those for the study since the last assessment, and, if yes, how many classes they attended.

**Physical activity and sedentary behavior.** To assess child physical activity, caregivers will be asked the number of days in past week the focal child was active for at least 60 min [92], and two questions about the amount of time the child “typically” spent playing outdoors in the last month – one related to weekdays and the other related to weekend days [93]. To assess sedentary behavior, caregivers will be asked to report the number of hours on an average school day that the focal child watches television, plays video games or computer games, or uses a computer for something that is not school work [92]. Questions drawn from the Middle School Youth Risk Behavior Survey [92] will be adapted for caregiver-report and to an online format.

**General health status.** Caregivers will be asked to report their general health and the general health of the focal child from five ordinal response options using questions from the NHANES converted to an online format [94]. They also will be asked to report how much time they (the adult) have spent outside per day between 10 am and 4 pm.

**Costs associated with food purchasing and preparation.** Caregivers will be asked 12 questions to assess (1) cooking supply costs; (2) grocery shopping expenses; (3) time and travel costs associated with shopping at the grocery store; (4) time spent planning meals; (5) time spent cooking meals; and (6) any costs associated with “eating out” or purchasing prepared meals and snacks not included in the reported grocery costs.

**Costs associated with CO-CSA participation (specified assessments only).** Costs associated with CO-CSA participation, including the time and financial costs associated with program participation and related food preparation behaviors, will be assessed in the fall each year the household is assigned to receive the CO-CSA. Questions will cover (1) employment and wages; (2) costs associated with travel to and from the CSA pick-up site and the F3HK healthy eating classes, including childcare costs, if applicable; (3) time spent at the pick-up site and healthy eating classes; and (4) the value of contributions to program success (e.g. volunteered time or donated supplies). Questions pertaining to the healthy eating classes will be asked only in the years and to the groups that the classes are offered.

**Kitchen inventory (baseline only).** At baseline, caregivers will be asked about ownership of 14 common kitchen implements (paring knife, chef’s knife, cutting board, spatula, cooking spoons, vegetable peeler, salad spinner, mixing bowl, frying/sauté pan, storage container, saucepan, colander, slow cooker, and food processor), how commonly they are used to prepare, cook, and store fresh fruit and vegetables, and their quality. For those that they do not own, caregivers will be asked which would make preparing, cooking, and storing fresh fruit and vegetables easier.

**Household composition and demographics.** At baseline, all adult participants will compete questions drawn from the American Community Survey [95] and BRFSS [96] about household composition (age, gender, and relationship of all household members) and demographic characteristics (race/ethnicity, education, employment, and household income). Questions and response options will be modified to an online format and simplified, where needed. Adult participants also will report whether anyone in the household smokes. Caregivers will be asked to provide the name, age, and gender of the focal child and the number of days in a typical week that he or she lives with the respondent.

On follow-up questionnaires, participants will be asked to update household composition, education, employment, income, smoking status, and days that child lives with respondent.

**CSA participation (outside of/in addition to the research study).** At each follow-up online survey, study participants will be asked if they participated in a CSA (not part of the intervention) during the CSA season since the last assessment.

##### Process evaluation

**Participant, educator, and performance site coordinator-level process evaluation.** A process evaluation will assess intervention dose delivered, dose received, and fidelity [97]. “Dose delivered” will be determined based on CSA share pick-up and lesson attendance. Participating farms will provide data on CSA share pick-up. Participants will complete an identifiable, paper-based questionnaire after each of the nine lessons to report their utilization of CSA share contents and their perceived utility of lesson components (“dose received”). Utilization also will be determined through a question on the fall online questionnaire which asks the portion of the share usually eaten. For households that report that they did not usually eat all of their share, follow-up questions related to preservation, spoilage, and food sharing will be asked. “Fidelity” will be assessed via post-lesson educator questionnaires and site coordinator quality assurance (QA) visits. Educators will report their fidelity to the written curriculum and their perceptions of the lesson’s acceptability, feasibility, and adaptability. Performance center coordinators will conduct two QA visits to each community during Years 2 and 3 and will audit that week’s CSA share pick-up and lesson facilitation. Audits will be used to collect information on the functionality of pick-up sites and CSA share contents. Class observations will be used to supplement educator’s reporting of lesson fidelity and to assess educators’ exhibition and use of qualities and techniques deemed important for adult education: preparedness, adaptability, knowledge, facilitation of group cohesion, respect, and promotion of a safe environment, immediacy of content and engagement. Following the CSA season, participant focus group discussions and educator interviews will be conducted to explore and elaborate upon findings from the above-described questionnaires.

**Program cost assessment.** F3HK program administration costs will be assessed via an 8-question questionnaire completed by performance center coordinators and relevant study personnel at the end of each CSA season. The questionnaire will assess the following cost categories: salaries, wages, and benefits; facilities and utilities; equipment, supplies, and travel; and staff training. These data will be used with the “costs associated with CO-CSA participation” data to determine the total value of resources used during administration of and participation in the F3HK program.

**Non-participation assessment.** Brief phone interviews including quantitative and qualitative questions will be used to understand the reasons eligible families choose not to participate in F3HK and the reasons why enrolled families cease participation (defined as missing four or more share pick-ups).

#### Unexpected events reporting procedure

Our unexpected events reporting procedure will require that the coordinating center be contacted any time a research staff member, nutrition educator, farmer, or research participant reports or observes a research participant or child experience an injury (e.g. a cut or fall) during the CO-CSA pick-up or education curriculum delivery, or that something occurs that could compromise participant privacy. The Principal Investigator (RAS) determines whether this event was a serious adverse event and, if so, the Cornell IRB will be notified within five business days or, if not, the event will be included on the normal reporting schedule.

#### Protocol amendments

The study protocol is consistent with the SPIRIT guidelines for clinical trials [98] (Additional file 2). Any protocol amendments will be developed with input from all co-investigators, and will be reviewed and approved by the University of Vermont and Cornell University IRBs prior to implementation. A written protocol document will be updated as needed and stored on a secure drive to which only members of the research team will have access.

#### Data management and analytic plan

##### Data management

Most data will be entered online directly by participants. All information collected will be kept private and confidential, and stored on a secure computer to which only members of the research team will have access. Participant’s names and all personal identifiers will be removed from data before analysis. All personal identifiers will be encrypted and password protected.

##### Analysis of primary and secondary intervention outcomes

Intent-to-treat analyses will allocate all participants to initial random assignment group for measurement at Year 2 baseline, Year 2 fall assessment, and Year 3 spring assessment. Subsequently, Group 2 will be considered to have received the intervention for measurement at Year 3 fall assessment, Year 4 spring assessment, and Year 4 fall assessment. Likewise, Groups 3 and 4 will maintain initial random assignment for measurement at Year 3 spring assessment, Year 3 fall assessment, and Year 4 spring assessment, but then Group 4 will be considered to have received the intervention for Year 4 fall assessment.

Change variables for linear outcomes (Table 4) will be created by subtracting the baseline from the measurement at the end of the CSA season, as well as during subsequent measurement points. Multilevel linear models with fixed and random effects will be used because observations are repeated measures for participants nested within farm communities. Models also will include intervention group status, baseline measures of outcome variables, and relevant covariates. Additional models will control for CO-CSA and education dose received. Multiple imputation will be used to estimate partial missing data.

##### Process evaluation

Logistic regression models will be used to assess whether differences in intervention implementation across sites (via “fidelity” measures) and differences in experiences between participants (via “dose delivered” and “dose received” measures) influence participants’ outcomes. Qualitative assessments of participants’ and educators’ experiences will be used to understand factors associated with dose and fidelity at each site. Data on non-participation will be analyzed using the Five Dimensions of Access framework [99]. Results will inform the use and refinement of the curriculum and CO-CSA model for future community-based nutrition interventions and will be helpful for CSA farmers interested in incorporating the CO-CSA model into their business operations.

##### Cost-effectiveness analysis

Using the data collected from performance center coordinators, a *cost analysis* will first be conducted to identify and measure the direct, tangible costs of the resources used in administration and implementation of the F3HK program. This analysis will be done from both the program perspective and the broader societal perspective. Analysis from the program perspective will focus on costs directly incurred during preparation for and implementation of the various components of the F3HK program, including the monetary value of in-kind contributions. Analysis from the societal perspective will estimate all costs incurred and health effects obtained as a result of the program, including estimation of costs and effects experienced beyond the participant and program perspectives [100].

A preliminary *cost-effectiveness analysis* (CEA) then will be completed, which will build on the cost analyses conducted from the broader societal perspective and will include opportunity costs experienced by participants (i.e. time at CSA site or education class that could have been used in other valued ways). We will use the human capital method and value participants’ time based on wage rates [101]. We will estimate the value of participants’ time using income data collected from participant questionnaires and national average wage rates from the Bureau of Labor Statistics [102]. An incremental cost-effectiveness ratio (ICER) will be calculated by (1) subtracting the cost of the F3HK program (as determined by the cost analysis) from the cost of the alternative (no program, $0); (2) subtracting the change in fruit and vegetable intake from baseline to late-season in the control group from the change in fruit and vegetable intake during that same interval in the intervention group, as measured by the FVS; and (3) dividing the program cost by the difference in the change in fruit and vegetable intake between the control and intervention groups. This ratio will thus provide an estimate of the program cost per unit change in fruit and vegetable intake. Epidemiologic data will be used to estimate the impact of changes in fruit and vegetable intake on life expectancy and quality, or Quality Adjusted Life Years (QALYs) [101], which will allow for a comparison of cost-effectiveness between F3HK and other programs aimed at improving the public’s health.

### Component 5: Economic impact on farmers and communities

#### Data collection and outcomes

We will assess the economic impact of the CO-CSA program at the farm level and at the community level. Measuring the profitability of CSA programs for farmers is complex [39, 40, 44, 47], and measuring the economic impact of a cost offset modification to farmers’ CSA programs presents additional challenges, as CO-CSA participants likely represent a small sub-section of the CSA membership of each farm. We will measure the economic outcomes of the CO-CSA for farms using in-depth interviews with farmers participating in the randomized trial (component 4). The questions in the interview guide will be used to assess the economic impact of adding cost offset shares to the farmers’ CSA. These questions will mostly be qualitative and will focus on changes to revenue stream, changes to profitability, impact on consumer base, and changes implemented on the farm (e.g. changes to the distribution model, acceptance of SNAP benefits, produce grown).

To estimate the economic impact of CO-CSA programs on local communities, we will use the input-output economic model (IMPLAN) and augment it with additional contextual data. While IMPLAN is one of the most commonly used models to conduct economic impact studies, researchers have found that the model does not fully capture the impacts of smaller, diversified farms and other small-to-medium scale operations that frequently participate in the localized food system [103–105]. Often, farmers selling to local and regional markets spend more money locally and spend it differently than “average” farms in the agricultural sector [106]. As a result, the economic impact of these farmers is different that the impact of the default agricultural sector in IMPLAN. To address the limitations of the default IMPLAN agricultural sector, we will customize the model using quantitative data collected from the participating farms at the time of the in-depth interviews. Questions will focus on producers’ annual sales, payroll, number of employees, operating expenses, and expenditure patterns such as input and services purchased and location of these purchases.

#### Economic analysis

Interviews with farmers will be conducted by trained study personnel at the end of Year 2 and will be audio-recorded and transcribed verbatim. The economic analysis will be conducted in two steps. First, qualitative data related to economic impact of the CO-CSA program at the farm level will be coded and analyzed using the content analysis approach [107] with the support of qualitative data analysis software. Second, economic data collected from farmers will be used to create a CSA farm sector in IMPLAN following procedures recommended in a recent USDA toolkit on assessing the economic impact of local food system initiatives [49]. We will extrapolate the CSA farm sector in each state using the data collected from farmers and the number of farms that have a CSA program based data from the 2012 Census of Agriculture. Once the CSA farm sector in IMPLAN has been created, we will calculate potential economic effect in each state under various scenarios such as the current economic contribution of the CSA farms to the state economies and the potential economic impact of the CSA farms sectors to state economies if the number of low-income participants increase through CO-CSA programs.

### Component 6: Develop business plans for long-term sustainability of CO-CSAs

Expanding markets to include low income families in a sustainable manner, without loss of profitability, is a key goal of this project. The research team will support this effort by (1) offering CSA farmers online resources informed by formative evaluation (component 1); (2) holding regular interactive webinars and online chats for farmers participating in the randomized trial (component 4) for peer-to-peer networking and problem-solving; and (3) convening the advisory group of agribusiness experts to provide technical assistance, provide feedback as the online resources are being developed, and host webinars or chats.

Beginning in Year 2, we will host chat sessions for farmers participating in component 4 (randomized trial) that will allow them to share experiences, discuss challenges, and brainstorm solutions in real time as they are implementing the first year of the F3HK program. Notes from these chat sessions will be incorporated with formative evaluation (component 1) results at the end of each season in order to inform improvements to component 4 (randomized trial).

We will also develop a toolkit with business support tools and resources based on findings from components 1, 2, and 5 (formative evaluation, longitudinal analysis, and economic analysis), along with an environmental scan of online resources. The toolkit will include a menu of strategies for subsidizing 50% of cost-offset shares. Possible strategies include having full-price members pay extra to cover the subsidy; grants from government agencies, businesses, or non-profits; community fundraisers (e.g. harvest dinners); or a combination of these approaches. The toolkit will include a summary of findings concerning the cost threshold full-price CSA members are willing to pay to subsidize others. The tool kit will be disseminated online through the project website, through cooperative extension and other appropriate channels beginning in Year 3.

The toolkit will support farmers participating in the randomized trial (component 4) as they begin developing business plans that will allow them to sustain the CO-CSA program in a manner which best suits their local context. Development of long term plans also will be supported by trainings that occur as part of an annual interactive webinar featuring extension agents, agripreneurs, and others with expertise in running CO-CSAs, to be held in Years 3 and 4. This component will be evaluated through online questionnaires and capture data related to overall reach. We will work with project partners to disseminate webinars.

### Component 7: Develop student education modules related to food systems and obesity prevention

An important component of the USDA’s Agriculture and Food Research Initiative (AFRI) call which funded this project is the inclusion of training opportunities for the “next generation of educators and scientists” in the area of childhood obesity and food systems research. While some instructional materials exist which relate to the potential benefits and desirability of food systems approaches to addressing health promotion for consumers and economic opportunities for farmers, there is a dearth of teaching materials that synthesize the emerging evidence of impact on both consumers (health) and producers (economic opportunity). To contribute to this relatively new area of teaching in higher education, we will conduct a review of the literature as well as existing instructional materials to determine important content area and what is currently addressed. Informed by this work, we will develop and disseminate materials for undergraduate and graduate level training that will assist course instructors with teaching evidence-based intervention strategies and research methods. This will include a case study approach, creating original modules to highlight relevant research projects that illustrate some of the challenges and opportunities that will likely be faced by emerging researchers and educators focusing on the nexus of childhood obesity and sustainable food systems.

### Dissemination

A committee has been established to review and approve all study dissemination materials, defined as abstracts, posters, manuscripts, and other products intended for internal or external dissemination that include data or descriptions of processes from work conducted as part of the project (Additional file 3). Authorship will be determined in line with the guidelines of the International Committee of Medical Journal Editors [108]. Datasets for this project will be made available on reasonable request.

## Discussion

Important disparities in diets and nutrition-related disease persist. Socioeconomically disadvantaged groups have lower intake of fruit and vegetables [15, 21, 22] and poorer diet quality [109], as well as higher risk of overweight, obesity, and a number of other chronic health conditions [7, 19]. Establishing healthy behaviors at an early age is important since they track between childhood and adulthood [110]. Dietary behaviors are determined by diverse environmental and individual factors, including access to acceptable nutritious foods and nutrition knowledge, attitudes, and beliefs. Approaches that address multiple constraints to healthy eating faced by children from poor families are needed in order to more effectively reduce current and future health disparities.

This integrated and innovative project has the potential to address some of the underlying determinants of poor diets among socioeconomically disadvantaged groups and, in so doing, positively affect fruit and vegetable intake, weight maintenance, and local agricultural economies. It will contribute new evidence about how local foods initiatives can more effectively meet the needs of low-income households and will produce information and resources that will help farmers and public health advocates across the nation develop and sustain CO-CSA programs while training the next generation of researchers.

## Additional files

Additional file 1: Cost Offset Community Supported Agriculture (CO-CSA) Research Intervention Consent﻿ Form. (DOC 150 kb)

Additional file 2: SPIRIT 2013 Checklist: Recommended Items to Address in a Clinical Trial Protocol and Related Documents. (DOC 123 kb)

Additional file 3: Publication, Presentation, and Dissemination Guidelines: *Innovative Community Supported Agriculture (CSA) Cost-Offset Intervention to Prevent Childhood Obesity and Strengthen Local Agricultural Economies*. (DOCX 69 kb)

## Abbreviations

AFRI: Agriculture and Food Research Initiative
AMPM: Automated Multiple-Pass Method
ASA24: Automated Self-Administered 24-h
BMI: Body mass index
BRFSS: Behavioral Risk Factor Surveillance System
BSQ2: Beverage and snack questionnaire 2
CEA: Cost-effectiveness Analysis
CO-CSA: Cost-offset community supported agriculture
CSA: Community supported agriculture
EBT: Electronic benefits transfer
EFNEP: Expanded Food and Nutrition Education Program
F3HK: Farm Fresh Foods for Healthy Kids intervention
FPL: federal poverty level
FSSM: Food Security Survey Module
FVS: National Cancer Institute’s All-Day Fruit and Vegetable Screener
ICER: Incremental cost-effectiveness ratio
IMPLAN: input-output economic model
IRB: Institutional Review Board
NC: North Carolina
NHANES: National Health and Nutrition Examination Survey
NOFA-VT: Northeast Organic Farming Association of Vermont
NY: New York
QA: Quality assurance
QALY: Quality adjusted life year
SNAP: Supplemental Nutrition Assistance Program
SNAP-Ed: Supplemental Nutrition Assistance Program Education
US: United States
USDA: United States Department of Agriculture
VT: Vermont
WA: Washington
WIC: Special Supplemental Food Program for Women, Infants, and Children
YRBSS: Youth Risk Behavior Surveillance System
