# Supplementary material for: Farm Fresh Foods for Healthy Kids (F3HK): An innovative community supported agriculture intervention to prevent childhood obesity in low-income families and strengthen local agricultural economies
Source: BMC Public Health. 2017 Apr 8;17:306. doi: 10.1186/s12889-017-4202-2 (PMC5385092; doi:10.1186/s12889-017-4202-2)
Supplement: Supplementary file 1 — Cost Offset Community Supported Agriculture (CO-CSA) Research Intervention Consent﻿ Form. (DOC 150 kb) [file 12889_2017_4202_MOESM1_ESM.doc]

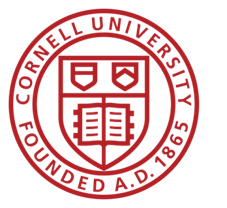
 <*insert logo for local research partner institution*>

**Cost Offset Community Supported Agriculture (CO-CSA) Research Intervention**

**Principal Investigator: Co-Principal Investigator:**

Rebecca A. Seguin, PhD, CSCS *<* ***insert name and***

Division of Nutritional Sciences ***contact information for local co-PI****>*

Cornell University

Ithaca, NY 14853

607.255.8250

[rebeccaseguin@cornell.edu](mailto:rebeccaseguin@cornell.edu)

**Project Summary**

Community Supported Agriculture (CSA) is one way to increase access to fresh fruits and vegetables. In a CSA, people pay for a ‘share’ of a farmer’s crops (sometimes called a produce box) and get fresh vegetables and fruit weekly. Some farms offer CSAs at a reduced price, called a “cost offset” CSA or CO-CSA. The aim of this research study is to better understand what is eaten by people who participate in both a CO-CSA and healthy eating classes focused on cooking the produce in the CSA share. This research is funded by the US Department of Agriculture (USDA).

This study will take place from 2016-2018. Research participants must take part for all three years, including the purchase of a reduced price CO-CSA share for two years and classes for one year. Families will be assigned by chance to one of two groups: group 1 will purchase the CO-CSA share and participate in classes in Summer 2016; and group 2 will get no CO-CSA in 2016, but then will purchase the CO-CSA share plus classes in Summer 2017.

**To be eligible**, you must:

- be 18 years of age or older;
- speak and read English;
- be the parent or legal guardian of at least one child in the household between 2 and 12 years old;
- consent for one of the above children to participate in the study, and the child must also be willing to participate;
- have access to a computer from which you can complete on-line data collection;
- have an active e-mail account or be willing to create an e-mail account;
- have not participated in a CSA in the past five years;
- be enrolled in (or have a family member in your household enrolled in) SNAP <**insert name of state SNAP program**>, WIC, or Head Start; OR, have a household income below 185% of the federal poverty level;
- be willing to purchase a cost-offset CSA share; and
- make a deposit on the CSA share within one week of enrollment.

**What we will ask you to do**

1. **How this CSA works.** The [**insert farm name**] CSA runs for [**number**] weeks, from roughly [**date**] to [**date**]. The study will pay half of the cost of the CSA share to the farmer at the beginning of the season. As a member, you will be responsible for showing up at [**the farm/pick-up site**] each week **to pay for and pick up your produce share**. Variety and quantity of produce will vary, and there may not be a choice of items. You will generally receive [**weight/volume/items**] of {**vegetables/fruits**}.

*<If there are share size options, please name each one and include a) an appropriate size statement, b) full price CSA for that share, c) CO-CSA subsidized price for that share.>*

1. **You can pick-up the CO-CSA share** at *<Insert detailed information regarding pick-up, including location(s) for the farm. Do not include any drop-off sites as participants will need to make a weekly balance payment.>*

*<If applicable, add text that differentiates sites where SNAP/EBT can be used from sites where only cash can be accepted.>*

You may pick any location we offer; however, we can only accept SNAP/EBT at **_____** location. You may arrange for someone else to pay for and pick up your share [**or you may place your share on hold; *insert only if applicable***] if you cannot.

1. **Your weekly cost** for the CO-CSA share is listed above. You must pay this reduced price every week at pick-up or you will not receive any produce that week.

3a. **You can pay with SNAP/EBT or [cash/check/credit/debit/etc. depending upon farm capabilities].** To pay with SNAP/EBT or credit/debit card, you must have your card with you at the time of payment.

3b. **A deposit of $___ is required** within one week after study enrollment. You can make your deposit by mailing a check or money order to your CSA farm in an addressed, stamped envelope that we will provide to you. This deposit cannot be paid with SNAP benefits. Your deposit will be refunded to you at the end of the CSA season, if you make all weekly payments. If you miss one payment, you will forfeit half of your deposit. If you miss more than one payment, you will lose your entire deposit.

**If you are assigned to group 1, you must pay your deposit within 7 days of enrollment or you will be removed from the study, will not be able to purchase the CO-CSA, and will receive no kitchen tools.**

**If you are assigned to group 2, you will pay your deposit in 2017.**

1. **Healthy eating classes** will help you learn how to prepare the produce in your CSA share. Participants are expected to attend 9 CSA-tailored healthy eating classes. Three classes will be held in the spring, three in the middle of the summer, and three in early fall. Classes are for parents, but children are welcome. The classes will be scheduled by the educator for days, times, and locations that are convenient for the most participants.
2. **Free kitchen tools** will be provided to help you in preparing your CSA produce. You can select 2 to 4 of the following items: food processor, crockpot, stockpot, large cutting board, salad spinner, reusable grocery bag, and chef’s knife. Participants assigned to group 1 will choose their kitchen tools in 2016, and those assigned to group 2 will choose items in 2017. Kitchen tools will be mailed directly to your home.
3. **Today, as part of enrollment in the research study**, we will ask you to provide two types of information.

6a. **Scanning of** **the palm of your hand** will be conducted by trained research staff. We will use a harmless device that measures the amount of carotenoids (a substance in fruits and vegetables) present in the skin. You will be compensated $10 for participation in scanning.

6b. **Completion of an on-line survey** on a tablet computer; or, you may have completed this survey on-line before today. This survey asks about your household, cooking and eating habits, your kitchen tools, your child’s physical activity, your income and expenses, and information about foods and drinks consumed by you and your child. Depending upon the age of your child, the survey may suggest that you have your child help to answer questions about their food and drink. The survey will take approximately 60 minutes to complete. We will provide snacks for you and your children while you complete this survey. You will be compensated $25 for completion of this survey.

**If you do not complete this survey, you will be removed from the study, will not be able to purchase the CO-CSA, and will receive no kitchen equipment.**

1. You also will be asked for **more information** throughout the three years of the study.

7a. **Scanning the skin on** **the palm of your hand** will be requested three more times. You will be compensated $10 on each date that you participate in scanning.

7b. **Completion of a similar on-line survey** will be requested five more times. All surveys will be completed on-line and take approximately 30 minutes on a computer, or 60 minutes on a tablet. You will be e-mailed when it is time to complete an on-line survey. Depending upon the age of your child, the survey may suggest that you have your child help to answer questions about their food and drink. Generally, you will be given two weeks to complete the survey. You will be compensated $25 for each survey that you complete.

If you complete three surveys in a row, you will also receive a $25 bonus payment. For example, if you received and responded to surveys (1) at enrollment, (2) in September 2016, and (3) in March 2016, you would have received $25 each time you completed one of those surveys. Since you completed all three, you would also receive a $25 bonus in March 2016. However, if you did not complete any one of those surveys, you would not receive a bonus in March 2016.

7c. **A focus group** will occur toward the end of your first summer of CO-CSA participation (either 2016 or 2017 depending upon group assignment). We will ask you to participate so that we can learn more about your experience with the CO-CSA and the produce you received. The time and location of this focus group will be scheduled closer to that time in order to make it convenient for most participants. You will be compensated $25 for your participation in this focus group.

7d. **A completion bonus can be earned** for consistent participation in all of the data collection activities described above. Adult participants who complete all data collection activities across all the three years of the study (6 surveys, 4 skin scans, and 1 focus group) will receive a bonus of $100.

1. **On-line dietary reports of ALL the food and drinks consumed by your child** will be requested for three separate days (for example, Wednesday, Thursday and Saturday). You will login to the National Cancer Institute’s online ASA24 program on three separate days, and each time you will report all the food and drinks consumed by your child on the previous day. You will need to use a computer (not a phone or tablet) with a high-speed internet connection (not dial-up). If you complete all three reports by the deadline given, you will receive $50 compensation. If you only complete one or two reports, you will not be paid. During the study, you will be asked to complete a total of four three-day dietary reports for your child. Each time, you will be compensated $50 if you complete all three days. Over the course of the study you may be compensated a total of $200 for these reports.
2. **Total compensation for completion of all data collection activities outlined below is $615.**

Compensation will be given as cash in-person or Visa gift card mailed to the participant’s home for on-line activities.

| **Time** | **Information requested** | **Adult Compensation** | | **Maximum Total to Date** |
| --- | --- | --- | --- | --- |
|  |  | **Regular** | **Bonus*** |  |
| Enrollment (Spring 2016) | Parent survey, may include child | $25 |  | $25 |
|  | Scan parent’s hand | $10 |  | $35 |
|  | ASA24 3-day reports, may include child | $50 |  | $85 |
| Sept 2016 | Parent survey, may include child | $25 |  | $110 |
|  | Scan parent’s hand | $10 |  | $120 |
|  | ASA24 3-day reports, may include child | $50 |  | $170 |
|  | Focus group | *Group 1 only*  $25 |  | $195 |
| March 2017 | Parent survey, may include child | $25 | $25 | $245 |
| Sept 2017 | Parent survey, may include child | $25 | $25 | $295 |
|  | Scan parent’s hand | $10 |  | $305 |
|  | ASA24 3-day reports, may include child | $50 |  | $355 |
|  | Focus group | *Group 2 only* $25 |  | (only participate in one focus group) |
| March 2018 | Parent survey, may include child | $25 | $25 | $405 |
| Sept 2018 | Parent survey, may include child | $25 | $25 | $455 |
|  | Scan parent’s hand | $10 |  | $465 |
|  | ASA24 3-day reports, may include child | $50 |  | $515 |
| **COMPLETION BONUS**, *if all survey and measurement information is provided every time (does not require all ASA24 daily reports)* | | | $100 | $615 |
| **MAXIMUM COMPENSATION *across 3 years*** | | **Adult = $615** | | |

** If three surveys in a row have been completed up to and including that time point, participants will receive the bonus in addition to the regular compensation.*

**Risks and discomforts**

**We do not anticipate any risks to you from participating in this research.** Skin scanning is harmless and will be conducted by trained research staff. We anticipate that your completion of on-line surveys and dietary reports presents no greater risk than everyday use of the Internet.

**Benefits**

**The probable benefits to you of participation in this research are improved access to fruits and vegetables, and the opportunity to increase knowledge and skills in the preparation of seasonal produce. The future indirect benefits include a better understanding of how to help people access affordable fruits and vegetables, and how to sustain local farms.**

**Privacy, Confidentiality, and Data Security**

Any information that you provide to us will be kept private and confidential, and stored on a secure computer to which only trained researchers have access. We anticipate that your participation in on-line surveys presents no greater risk than everyday use of the Internet. Your name and all personal identifiers will be removed from all data before it is analyzed. No names will ever be used in reporting results from this survey.

Throughout this research study, we will communicate with you through e-mail. Please note that email communication is neither private nor secure. Though we are taking precautions to protect your privacy, you should be aware that information sent through e-mail could be read by a third party.

**Taking part is voluntary**

In order to participate in this research study, you must 1) complete the on-line enrollment survey, and 2) if assigned to group 1, pay the indicated deposit to the CSA farm within 7 days of enrollment.

Your participation in other activities is voluntary. You may refuse to participate before the study begins, skip any questions that make you feel uncomfortable, or withdraw from the research study at any time. There will be no penalty for withdrawing from the study, and no effect on any compensation earned before withdrawing. If you do not wish to participate in this research study, it will not affect your relationship with *<****insert name of local research partner institution****>* or with Cornell University.

**If you have questions**

**Please ask any questions that you have now.** The main researchers conducting this study are Rebecca Seguin, a professor at Cornell University, and *<****insert name of local co-PI****>.* If you wish to contact either of them, their phone numbers and e-mail addresses are listed at the top of this form. If you have any questions or concerns regarding your rights as a subject in this study, you may contact the Cornell University Institutional Review Board (IRB) for Human Participants at 607-255-6182 or access their website at [http://www.irb.cornell.edu](http://www.irb.cornell.edu/). You may also report your concerns or complaints anonymously through Ethicspoint online at [www.hotline.cornell.edu](http://www.hotline.cornell.edu/) or by calling toll free at 1-866-293-3077. Ethicspoint is an independent organization that serves as a liaison between the University and the person bringing the complaint so that anonymity can be ensured.

You will be given a copy of this form to keep for your records.

**Statement of Consent**

I have read the above information, and have received answers to any questions I asked. I consent to take part in the study.

Your Signature Date ______

Your Name (printed)

Signature of person obtaining consent Date

Printed name of person obtaining consent

*This consent form will be kept by the researcher for five years beyond the end of the study.*

**Parental Consent for Child Intervention Participation**

**What we will ask your child to do**

1. **Today, as part of enrollment in the research study**, we will ask your child to participate in the activities described below.

1a. **Measurement of your child’s height and weight** will be taken by trained research staff behind a privacy screen. Your child will be compensated $10 for the measurement of his/her height and weight.

1b. **Scanning of your child’s palm** will be conducted by trained research staff. We will use a harmless device that measures the amount of carotenoids (a substance in fruits and vegetables) present in the skin. Your child will be compensated $10 for participation in scanning.

1c. **Help with** **an on-line survey**; or, your child may have helped you complete this survey on-line before today. Depending upon the age of your child, the survey may suggest that you have your child help to answer questions about their food and drink. The section on your child’s food and drink will take approximately 10 minutes to complete.

1. Your child also will be asked to **provide more information** throughout the three years of the study.

2a. **Measurements of your child’s height and weight** will be taken three more times. Your child will be compensated $10 on each date that we measure his/her height and weight.

2b. **Scanning of your child’s palm** will be taken by trained research staff three more times. Your child will be compensated $10 on each date that we scan his/her hand.

2c. **Help with a similar on-line survey** may be requested five more times depending upon the age of your child.

2d. **Help with on-line dietary reports of ALL the food and drinks consumed by your child** may be requested. During the study, you will be asked to complete a total of four three-day dietary reports for your child. Depending upon the age of your child, the on-line dietary report may suggest that you have your child help to answer questions about their food and drink.

**Risks and discomforts**

**There is a small chance that your child could feel uncomfortable or embarrassed when weighed and measured. Trained research staff will perform measurements behind screens that protect your child’s privacy.**

**Benefits**

**There are no direct benefits from your child’s participation in this study.**

**Privacy, Confidentiality, and Data Security**

**We will protect your child’s information as described above. Only the researchers on this study will have access to any data.**

**Taking part is voluntary**

**Your child’s participation is voluntary. Your child may refuse to participate before the study begins, may skip any data collection activities that make him/her feel uncomfortable, or withdraw from the research study at any time.**

**Statement of Consent**

I have read the above information, and have received answers to any questions I asked. I consent for my child to take part in the study.

Your Signature Date ______

Your Name (printed)

Signature of person obtaining consent Date

Printed name of person obtaining consent

**Oral Assent Received (*children 7-12 years only*)**

Oral assent for participation in this study was obtained from my child.

Your Signature Date ______

Your Name (printed)

Signature of person obtaining consent Date

Printed name of person obtaining consent

**Child Assent for Intervention Participation – Information Sheet**

**What we will ask you to do**

1. **Today, as part of enrollment in the research study**, we will ask you to participate in the activities listed here.

1a. **Measurement of your height and weight** will be taken behind a privacy screen, and you will receive $10 for these measures.

1b. **Scanning of your palm** will be done using a harmless device that sends out a beam of light. If you like, I can show you using the palm of my hand now. You will receive $10 for this scanning.

1c. **Help with** **an on-line survey;** or, you may have helped with this before today. Your parent may ask you to help answer questions about the food you eat and the drinks you have. This will take approximately 10 minutes, and we will provide snacks while you help.

1. You also will be asked to **provide more information** throughout the next three years.

2a. **Measurement of your height and weight** will be taken three more times after today. You will receive $10 on each date that we measure your height and weight.

2b. **Scanning of your palm** will be done three more times after today, using this same device. You will receive $10 on each date that we scan your hand.

2c. **Help with** **a similar on-line survey** may be requested five more times. Your parent may ask you to help answer questions about the food you eat and the drinks you have. This will take approximately 10 minutes each time.

2d. **Help with on-line dietary reports of ALL your food and drinks** may be requested four times throughout the three-year study. Each time, your parent may ask you to help him/her answer questions about the food you ate and the drinks you had on three different days.

If you complete all 4 skin scans and 4 measures of height and body weight, you will receive a bonus of $20.

**Risks and discomforts**

**There is a slight chance that you may feel uncomfortable or embarrassed when weighed and measured. To make you feel more comfortable, we will take your height and weight behind a screen.**

**Benefits**

**There are no direct benefits to you from participating in this study.**

**Privacy**

**Only researchers on this study will know the information you share with us and they will keep all that information private.**

**Taking part is voluntary**

**Participation is voluntary. You may refuse to participate before the study begins, may skip any activities or questions that make you feel uncomfortable, or stop at any time.**

**Child Assent for Intervention Participation – Oral Assent Protocol**

- ALL CHILD PARTICIPANTS, 2-12 YEARS: Parental permission will be obtained and the parent will sign permission to have child participate in the project.
- CHILDREN 2-6 YEARS: Oral assent will be waived for children between the ages of 2-6 years.
- CHILDREN 7-12 YEARS: We will be obtaining ***oral assent*** from children between the ages of 7-12 years.

1. A trained researcher will slowly review the information sheet with the 7-12 year old child. After each activity, the researcher will ask the child if s/he has any questions.
2. After reading the information sheet to the child, the researcher will ask the child:
   1. Do you have any questions?
   2. Do you agree to take part in this study?
3. If the child agrees to take part in the study, the parent will sign the bottom of the parental permission form (page 9), indicating that oral assent for participation in the study was obtained from his or her child.
