# Supplementary material for: Farm Fresh Foods for Healthy Kids (F3HK): An innovative community supported agriculture intervention to prevent childhood obesity in low-income families and strengthen local agricultural economies
Source: BMC Public Health. 2017 Apr 8;17:306. doi: 10.1186/s12889-017-4202-2 (PMC5385092; doi:10.1186/s12889-017-4202-2)
Supplement: Supplementary file 3 — Publication, Presentation, and Dissemination Guidelines: Innovative Community Supported Agriculture (CSA) Cost-Offset Intervention to Prevent Childhood Obesity and Strengthen Local Agricultural Economies. (DOCX 69 kb) [file 12889_2017_4202_MOESM3_ESM.docx]

**Publication, Presentation, and Dissemination Guidelines:**

***Innovative Community Supported Agriculture (CSA) Cost-Offset Intervention to Prevent Childhood Obesity and***

***Strengthen Local Agricultural Economies* Project**

Policy Scope

This Policy for Authorship applies to all dissemination materials including abstracts, posters, manuscripts, and other products intended for internal or external dissemination that include information such as data or descriptions of processes from work conducted as part of the ***Innovative Community Supported Agriculture Cost-Offset Intervention to Prevent Childhood Obesity and Strengthen Local Agricultural Economies*** **Project**, herein referred to as the **CO-CSA Project**. Publications and other dissemination products are an important part of academic productivity and promotion and the process for determining authorship and content of such publications and other dissemination products needs to adhere to accepted ethical and integrity standards. The purpose of this policy is to provide such standards to guide authors while preparing and submitting scholarly publications or other dissemination products.

The Publications, Presentations, and Dissemination Committee (PPDC)

This committee consists of the study PI (Seguin, Chair) and co-investigator (Pitts, Co-Chair) as well as co-investigators Ammerman, Hanson, Kolodinsky, and Sitaker (PPDC members); they will have the responsibility of reviewing and approving study dissemination materials (as defined above). A dissemination product uses CO-CSA Project data (all or in part) and/or describes CO-CSA Project methods or processes and is intended for publication or presentation. There are three categories of dissemination products: (1) Non-peer reviewed educational materials or modules; (2) Non-peer reviewed products such as fact sheets or presentations for the public; and (3) Peer-reviewed, scientific products such as manuscripts or abstracts for national meetings.

For Category 1 products, the author of the product must submit the product to the CO-CSA Project Manager. The Project Manager will then notify all members of the PPDC and add the product to the agenda for the next bi-weekly Project meeting. The main goal of adding the product to the agenda for discussion is to notify all members of the research team that this module has been created, in order to avoid duplication. Educational modules will also be archived by the Project Manager, so that we can keep a record of the content and quantity of education modules created and presented.

For Category 2 products, the author must submit the product to the CO-CSA Project Manager, Leah Conner, via email. The Project Manager will notify Seguin and Pitts, who will then distribute the product to the appropriate team member for review. The product will also be discussed at the bi-weekly Project meeting. At least one PPDC member will review and approve the Category II product, which will be archived by the Project Manager upon approval.

For Category 3 products, the author must submit a proposal form to Project Manager, Leah Conner, via email. A general proposal form that must be used is at the end of this document. Incomplete proposals will not be reviewed and will result in delays. The project manager will notify the PPDC, send the proposal form to the entire group as an email attachment for the bi-monthly meeting PPDC discussion. During the bi-monthly PPDC discussion, a minimum of three members of the PPDC must approve the proposal form before the data request/proposal can move forward. If any PPDC member requests a major revision to the form (e.g., change in analysis plan), she can request this verbally during the meeting or in writing: either directly to the primary author or anonymously to the Project Manager within one week. Once the proposal form is approved and the data are released, the final paper must be submitted within six months of data release.

At least one CO-CSA co-investigator must sponsor (and co-author) any/all study dissemination materials. ***Note****: In the case of more than one sponsor, sponsors take equal responsibility in ensuring the quality, timeliness, and completeness of all proposals and completed works.*

Authorship Criteria

According to the guidelines of the International Committee of Medical Journal Editors (ICMJE), as revised in 2013, authorship credit must be based on the following 4 criteria:

1. substantial contributions to conception or design of the work, or the acquisition, analysis, or interpretation of data for the work; **and**
2. drafting of the work or revising it critically for important intellectual content; **and**
3. final approval of the version to be published; **and**
4. agreement to be accountable for all aspects of the work in ensuring that questions related to the accuracy or integrity of any part of the work are appropriately investigated and resolved.

Each author must be accountable for the parts of the work he/she has done. Each author must be able to identify which coauthors are responsible for specific other parts of the work and must have confidence in the integrity of the contributions of any coauthors. All those designated as authors must meet all 4 criteria for authorship, and all who meet the 4 criteria must be identified as authors. Those who do not meet all four criteria must be acknowledged.

Dissertation/Thesis Work Using Data from the CO-CSA Project

For dissertation or thesis-related work, the outlined guidelines/process applies. Students are encouraged to plan in advance and allow extra time, given that committee feedback is also needed. When the dissemination product proposal is submitted, committee members who are not CO-CSA investigators need to be proposed in advance and noted on the proposal form. If the full committee has not yet formed, the student and sponsor can request to amend the writing group at a later date, provided all authorship requirements can be met. Also, please note that CO-CSA data may not be used by students if the data relate to major CO-CSA papers in progress or planned if PPDC deems those data to be necessary for a future major paper. The list of planned major papers as well as active, approved papers will be generated by the CO-CSA investigators, but since major papers may emerge as the project progresses, the sponsor should send an inquiry about the general idea to the PPDC chair and co-chair prior to the student formally proposing the paper. The writing group is to take no action regarding the paper until the student has completed and defended the dissertation or thesis, provided this occurs in a reasonable length of time, to be determined by PPDC (for a dissertation, this is roughly one year, and for a thesis/masters paper, this is roughly six months). It should also be noted that unlike other writing groups without a dissertation or thesis where multiple authors may contribute to the analysis and writing of various sections, it is expected and understood that for student-led work, the student will complete the analysis and writing; the writing group’s role will be limited to providing feedback on the drafted manuscript. In addition, the student’s sponsor, not the student, will be the corresponding authors on submitted papers, although it is acceptable to have the student be the first author. The student's sponsor is to report the student's progress to the PPDC a minimum of once annually. The PPDC reserves the right to proceed with preparing a paper on the dissertation/thesis topic for publication through the activation of a writing group if the student has not submitted the paper within three months after the defense of the dissertation/thesis, and if the student’s sponsor has not requested to take over the paper due to lack of progress or interest from the student. After three months post-defense, if the dissemination product has not been submitted, the student’s sponsor must submit content, dataset, and data analysis files related to the student’s work back to the PPDC.

Primary Outcome Paper

The PI (R. Seguin) will take the lead in writing the first paper for the primary hypotheses outlined in the proposal. All co-investigators will be invited to contribute to this paper and be co-authors. This paper has priority for staff time and resources over all other potential publications.

Dissemination Product Proposals

A template of the dissemination product proposal form is included at the end of this document. Proposal forms must be completed in their entirety and by the individual proposing the specific dissemination product (manuscript, abstract, dissertation, thesis, education module, etc.). Dissemination product proposal forms must be emailed to the CO-CSA Project Manager, who will check the form for completion. Each proposal form will be added to the bi-monthly meeting agenda for discuss. For any dissemination product, anonymous feedback can be sent to the Project Manager, who will then send the feedback to the primary author. Other feedback can be discussed on the conference call, given adequate time for discussion. PPDC members (as many as are available) will review proposals between the bimonthly investigator team calls. Proposals will be discussed on the calls; feedback and/or approval will be provided to the lead contact person (if a student, it will go to both the student and the sponsor) within 5 business days. PPDC approval will be indicated by signing the bottom of the submitted form; submitters must send all materials in Word and leave adequate space for signature and date for the PPDC chair or co-chair to sign; if requested by a chair/co-chair, another PPDC member can sign. Datasets will be prepared and shared securely and put in queue based upon priority status and order received, determined by the study PI.

Joining a Writing Group

Upon approval, the product lead (or sponsor, if applicable) is required to circulate the approved proposal to all study co-investigators and team members within one week. Individuals who are able to make a **substantive** contribution to the product (e.g. manuscript, abstract, etc.) that is consistent with the authorship criteria stated above can request to be added by contacting the lead author/contact person with this request within one week of its circulation. The individual requesting co-authorship must include a description of what his/her contribution will be and/or his/her area of expertise. At the end of two weeks post-approval, the lead (or the sponsor, if applicable) will return to the PPDC a complete writing group list of participants—to include full name, mailing address, title, institution, phone number, and email address. A table template is provided for this purpose. ***Note****: being a project director/co-investigator does not automatically grant authorship; the process outlined must be followed by all individuals. Also, failure by any listed co-author to contribute (per authorship criteria) and/or to submit their feedback based upon the lead author’s timeline will result in a co-author being removed from the co-author list.*

Timeline to Submission

Timely submission of all materials is strongly encouraged. Once a proposal has been reviewed and approved, analyses must begin soon thereafter and writing assignments to writing group members should be assigned. If substantive progress has not been made toward completing and submitting the manuscript within six (6) months of data becoming available, another co-author listed on the manuscript approval form may petition the PPDC co-chairs to take the lead on its completion. The co-chairs will help to arbitrate the request with the original first author. If at any point a lead author knows he/she cannot meet the timeline or is no longer able to lead the dissemination product, he/she should contact the PPDC so that new leadership can be assigned. If timely progress is not being made and no co-authors want to lead the paper, the PPDC will brainstorm with the lead author on overcoming barriers to progress and try to find an agreeable solution. Ultimately, publication fees are the responsibility of lead author. There is money available in the grant budget to pay for open access journals. However, this funding is intended for main results and other priority papers that the team feels are important for open access.

Archiving CO-CSA Dissemination Materials

When any dissemination material resulting from full or partial use of CO-CSA data, materials, or methods is accepted for publication or presentation, the lead author (aka product lead) is responsible for sending the PPDC chair (Seguin) a copy of the **final** material, the citation, and other relevant details (specific to the type of material it is). The coordinating center will maintain all archives. Request from co-investigators for copies and/or requests from outside parties must be fulfilled by the coordinating center so that requests and dissemination activities can be appropriately tracked and shared with the Funder as needed.

Authorship Grievances

If there are researchers who believe they deserve authorship on any particular paper or presentation and the first author disagrees, or if there is a dispute about the order of authorship, the disagreement will be forwarded for appeal to the PPDC co-chairs, PI (Seguin) and co-investigator (Pitts), who will work with the conflicting team members to resolve the conflict. If agreement cannot be reached using mediation by the PPDC co-chairs, another co-investigator on the team uninvolved in the grievance will be chosen at random to help resolve the matter. It is the responsibility of the primary author, sponsor, and / or senior author to make ethical and field-specific decisions about authorship order.

CO-CSA Project Data Availability

All data that are part of CO-CSA Project will be maintained and made available to investigators by study coordinating center at Cornell University (PI: Seguin). Access to appropriate data files for investigators, staff, students, and post docs must be arranged using the proposal form to the PPDC.

Unpublished Data in Grant Applications or Contract Proposals

Investigators who seek to use CO-CSA Project data that have not been previously published but are needed for grant applications or contract proposals must have prior approval for use by the PPDC.

Publicity Policies

Publicity related to any findings being published from the CO-CSA project will be reported to the PI in advance of the actual publicity. Investigators that present findings to the media must take care to insure that data interpretations reported in press releases closely reflect and do not overstate the data presented. The coordinating center must be notified about upcoming press releases with sufficient lead time to alert and coordinate with the USDA press office.

All media materials and talking points must be reviewed and approved by the PPDC and USDA prior to distribution. These materials are assigned to 2 reviewers from the PPDC; they will expedite review to comply with deadlines, but authors are expected to provide adequate time for review, i.e. 10 business days, when possible. In the case of a press release being issued for a presentation based on a manuscript not yet accepted for publication in a peer review journal, a sentence must be included on the front page indicating the preliminary nature of the results.

Scientific Product Guidelines (manuscripts and abstract)

*General Guidelines.* The PPDC works to ensure consistency across CO-CSA publications with these guidelines:

- All publications must reference the main results paper, if published, and/or any other relevant CO-CSA products.
- Conclusions concerning individual outcomes must be presented in a way that considers the main outcome.
- Both hazard ratios and absolute rates must be presented when analyzing effects of the intervention.
- The prescribed acknowledgement section must be included. [See acknowledgements section.]

*Writing Clarity.* PPDC will consider the following when examining draft papers and proposals:

- Does the author identify a senior PI from CO-CSA to sponsor the paper?
- Does the paper’s topic overlap with existing literature? If so, do the authors reference those findings and discuss how they impact their current work?
- Confirm that tables and graphs are relevant and well-labeled.
- Is the writing clear and readable?

*Reporting of Race and Ethnicity Data.* CO-CSA Project publications must describe the demographics of the study population included in the analysis, or refer to another publication that describes the demographics. The demographics must include a listing by race/ethnicity (numbers and/or percentages). The following guidelines apply:

- The race/ethnicity subgroups (alternative nomenclature in brackets) that must be listed in alphabetic order are those by which participants identified themselves at enrollment, with some minor modifications: American Indian/Alaskan Native (American Indian or Alaskan Native); Asian/Pacific Islander (Asian or Pacific Islander); Black (African-American); Hispanic (Latino); White not of Hispanic origin; Unknown (not one of above).
- The unqualified use of the term “other” must be avoided.
- “Combining” these specific race-ethnic groups in the descriptive demographic tables is not allowed.

*Statistical Guidelines.* ***NOTE:*** *Different disciplines use different statistical conventions and we will defer to the first author to decide on the most appropriate statistical methods.* The following are general guidelines related to statistical issues:

- Two sided p-values must be used.
- Subgroup analyses must report number of subgroups and address the possibility of Type I error, by stating the number of comparisons that could be significant by chance alone.
- Stratified analyses must include assessments of interaction terms; if interaction terms are not significant, an interpretation of the stratified data should be agreed upon by the writing group and a justification for the interpretation presented to the PPDC along with the submitted manuscript. There may be limited power for testing interactions.
- Though priority papers focus primarily on nominal Confidence Intervals (CIs), issues of sequential monitoring and multiple testing must be considered. In some cases adjusted CIs need to be presented, while in others an acknowledgment of the potential for over-interpretation of the data will suffice.

Acknowledgment Policy

*Dissemination documents from the CO-CSA project* ***must*** *follow NIFA (shown verbatim below).*

NIFA ACKNOWLEDGMENT POLICY: Acknowledgment of USDA Support by NIFA

- When acknowledging USDA support in accordance with 2 CFR Part 415, grantees must use the following acknowledgment for all projects or initiatives supported by the National Institute of Food and Agriculture (NIFA): "This material is based upon work that is supported by the National Institute of Food and Agriculture, U.S. Department of Agriculture, under award number 2014-08347.
- Whenever practical, NIFA also expects that grantees use NIFA’s official identifier in publications, posters, websites and presentations resulting from their award. This identifier can be found at <http://nifa.usda.gov/resource/official-nifa-identifier>.
- In addition, all publications and other materials, except scientific articles or papers published in scientific journals, must contain the following statement: "Any opinions, findings, conclusions, or recommendations expressed in this publication are those of the author(s) and do not necessarily reflect the view of the U.S. Department of Agriculture."
- Press Announcements and Media Interviews Recommended awards must not be announced in any manner by a grantee until approval is obtained by NIFA’s communication office. This applies to press releases, newsletters, and press interviews, web or social media postings. In many cases, the Secretary of Agriculture may want to personally announce the award.
- NIFA grant awardees are requested to coordinate all public announcements with NIFA¿s Communication Staff at [CommunicationsStaff@nifa.usda.gov](mailto:CommunicationsStaff@nifa.usda.gov) (link sends e-mail). Simultaneous announcement by NIFA and the grantee are preferred.
- The awardee is responsible for acknowledging NIFA support during news media interviews that discuss work supported by NIFA.

**CO-CSA Dissemination Product Form for Categories 1, 2, and 3**

Category 1: Non-peer reviewed educational materials or modules.

1. Date of request:
2. Title:
3. Lead author’s name and contact information (include site affiliation):
4. Sponsor’s name and contact information (include site affiliation, if applicable):
5. Co-authors’ names and contact information (include site affiliation, if applicable):
6. Target event, or course (for a presentation or education/course module, list all details related to the presentation including organization, date, location, etc.):
7. Primary objective:
8. Timeline, please indicate date:
9. Special request (if specific data are needed):

Approval by PPDC

1. Date approved:
2. Signature indicating approval: ______________________
3. Document Approval #: ED#____________
4. Date final draft submitted for the archives.________________________

Category 2: Non-peer reviewed products such as fact sheets or presentations for the public.

1. Date of request:
2. Title:
3. Lead author’s name and contact information (include site affiliation):
4. Sponsor’s name and contact information (include site affiliation, if applicable):
5. Co-authors’ names and contact information (include site affiliation, if applicable):
6. Target event, or course (for a presentation or education/course module, list all details related to the presentation including organization, date, location, etc.):
7. Primary objective:
8. Timeline for presentation, please indicate date:
9. Special request (if specific data are needed):

Approval by PPDC

1. Date approved:
2. Signature indicating approval: ______________________
3. Document Approval #: PR#____________
4. Date final draft submitted for the archives.________________________

Category 3: Peer-reviewed, scientific products such as manuscripts or abstracts for national meetings.

1. Date of request:

2. For which of the following are you requesting review/approval or submitting final materials (select one):

- Manuscript proposal
- Manuscript final draft (for archiving)
- Abstract proposal
- Abstract final draft (for archiving)

3. Title:

4. Lead author’s name and contact information (include site affiliation):

Is this a thesis or dissertation project?

5. Sponsor’s name and contact information (include site affiliation, if applicable):

6. Co-authors’ names and contact information (include site affiliation, if applicable):

7. Target journal (list top 3 in order of preference) or intended conference (for submission of a final abstract or manuscript, list the full citation):

8. Primary research question(s)/hypotheses:

9. Data requested (note that all needed variables must be listed in advance, as this will be used to generate the dataset; failure to list all variables will results in delays in the analysis process):

| Variable/variable name | Source/Time Points |
| --- | --- |
|  |  |
|  |  |

10. Analysis Plan *(for quantitative and qualitative abstracts or manuscripts only)* you must: *1)* describe your statistical analysis plan; 2) name your analyst/statistician; and 3) provide drafts of all planned tables (with titles and column and row labels) and/or figures (with titles and descriptions).

11. Timeline (not applicable for submission of final products):

- Manuscripts are to be submitted for review within 6 months of receiving the dataset. Please initial to affirm that you will adhere to this timeline:
- If for Conference Abstract, please indicate abstract due date:

Approval by PPDC

13. Date approved:

14. Signature indicating approval: ______________________

15. Date data request is met (if applicable): ___________________

16. Document Approval #:

MS#____________

AB#____________

17. Date final draft submitted for the archives.________________________

CO-CSA Writing Group Author List Submission *(to be submitted within 2 weeks of a product approval)*

*All of the following fields must be complete*

| Last name | First name | Title | Institution | Mailing Address | Phone number | Email address | Expertise/contribution |
| --- | --- | --- | --- | --- | --- | --- | --- |
|  |  |  |  |  |  |  |  |
|  |  |  |  |  |  |  |  |
|  |  |  |  |  |  |  |  |
|  |  |  |  |  |  |  |  |
|  |  |  |  |  |  |  |  |
|  |  |  |  |  |  |  |  |
|  |  |  |  |  |  |  |  |

**Appendix**.

Overall Philosophy of the PPDC:

- Encourage productivity -- ensure that data requests are translated into papers submitted in a timely fashion.
- Avoid duplicity – avoid reinventing the wheel for non-peer reviewed presentations, etc.
- Encourage collaboration –more minds are better than one.
- Streamline to avoid unnecessary hoop-jumping.
- Archive all products for future use and for funder’s records.

General Process and Framework:

Project manager will notify the PPDC and add to the bi-monthly agenda.

At least one PPDC member will review and approve the Category II product.

Project manager will notify the PPDC, add to the bi-monthly agenda, and archive.

Category II: Non-peer reviewed products such as fact sheets or presentations for the public.

Category I: Non-peer reviewed educational materials or modules.

Category III: Peer-reviewed, scientific products such as manuscripts, abstracts, or presentations for scientific / professional meetings.

**PPDC Team**

Chair: Seguin

Co-Chair: Jilcott Pitts

Members: Ammerman, Hanson, Kolodinsky, Sitaker

Submit Category I product form and final product to Project Manager, Leah Conner, via email.

Submit Category II product form and final product to Project Manager, Leah Conner, via email.

Upon approval, the Project manager will archive.

Once the proposal form is approved and the data are released, the final paper must be submitted within six months of data release.

Submit Category III proposal form to Project Manager, Leah Conner, via email.

Project manager will notify the PPDC, send the proposal form to the entire group as an email attachment for the bi-monthly meeting PPDC discussion.

If any PPDC member requests a major revision to the form (e.g., change in analysis plan), she can request this verbally during the meeting or in writing: either directly to the primary author or anonymously to the Project Manager.

During the bi-monthly meeting PPDC discussion, a minimum of three members of the PPDC must approve the proposal form before the data request can move forward.

**Addendum (02/08/2016)**

**PPDC addendum for ‘late-breaking’ opportunities**

**APPROVED 2/8/2016**

To seek provisional approval in order to take advantage of ‘late-breaking’ opportunities.

Late breaking opportunities are those for which the deadline for submission of an abstract, presentation or manuscript is in less than two weeks and the opportunity is not part of a regularly scheduled meeting with published/posted deadlines.

- Late-breaking opportunities can be sent to Leah Connor, including this addendum form with supporting documents.
- Leah will circulate the form and materials to the entire CO-CSA team, with a deadline for review of three days out.
- Late-breaking opportunities need approval from at least 3 co-PIs (not including the sponsor).

1. Date of request:
2. Date of deadline:
3. For which of the following are you requesting approval (select one):

- Abstract/presentation/poster proposal
- Manuscript proposal

1. Title:
2. Lead author’s name and contact information (include site affiliation):

Is this a thesis or dissertation project?

1. Sponsor’s name and contact information (include site affiliation, if applicable):
2. Co-authors

- List names and contact information (include site affiliation, if applicable):
- Verify that you have invited all co-PIs to join (or decline) participation

1. Full name of meeting, conference or journal, including date(s) and location
2. Primary research question(s)/hypotheses:
3. Data requested
4. General analytic approach (note: something as simple as “quantitative” is adequate for late breaking requests; additional details will be requested later)
